# Polymer coated gold nanoshells for combinational photochemotherapy of pancreatic cancer with gemcitabine

**DOI:** 10.1038/s41598-021-88909-x

**Published:** 2021-04-30

**Authors:** Mina Emamzadeh, George Pasparakis

**Affiliations:** 1 School of Pharmacy, University College London, London, WC1N 1AX UK; 2 Present Address: Department of Chemical Engineering, University of Patras, Patras, Greece

**Keywords:** Biomaterials, Nanoscale materials, Cancer, Drug delivery, Materials science

## Abstract

Pancreatic cancer is one of the most lethal malignancies with limited therapeutic options and dismal prognosis. Gemcitabine is the front-line drug against pancreatic cancer however with limited improvement of therapeutic outcomes. In this study we envisaged the integration of GEM with gold nanoshells which constitute an interesting class of nanomaterials with excellent photothermal conversion properties. Nanoshells were coated with thiol-capped poly(ethylene glycol) methacrylate polymers of different molecular weight via Au–S attachment. It was found that the molecular weight of the polymers affects the in vitro performance of the formulations; more importantly we demonstrate that the EC_50_ of nanoshell loaded GEM can be suppressed but fully restored and even improved upon laser irradiation. Our proposed nanoformulations outperformed the cytotoxicity of the parent drug and showed confined synergism under the tested in vitro conditions.

## Introduction

Pancreatic cancer is one the most aggressive human malignancies, with an extremely dismal prognosis due to absence of symptoms and lack of reliable screening tests for early diagnosis. The incidence of pancreatic cancer is almost equal with the mortality rate, with less than 5% 5-year survival^1,2^.

Gemcitabine (Gem) is currently the leading therapeutic for pancreatic cancer therapy, administered as monotherapy or in combination with other drugs in order to mitigate the problem of drug resistance development and to augment the therapeutic outcome^3^. However, Gem has a short plasma half-life (< 20 min) due to the rapid conversion to the inactive metabolite (2′,2′-difluorouridine) by cytidine deaminase which severely limits its efficacy^4^. Also, the deep-tumor delivery of Gem in pancreatic tumors is compromised due to desmoplasia^5^ comprising excessive accumulation of proteinaceous material with cytokines and stellate cells which prevents deep diffusion of the drugs at the center of the tumor site.

The use of hyperthermia in combination with chemotherapy^6,7^ constitutes a potent means to alleviate these obstacles as confined temperature increases exert multifarious effects on tumor physiology at the tumor and cellular level including the enhancement of blood/fluid (which augments the diffusion of the drugs in deep tumor areas accompanied by enhanced drug chemosensitization of cancer cells)^8^, increase of cell membrane fluidity (and permeability) by the transformation of the packed gel phase lipid layer to the less stiff liquid-crystalline phase^9^, fragmentation of the endoplasmic reticulum, reduction of ATP production^10^, heat-shock protein overexpression^11^ and ultimately, triggering of apoptotic caspase signaling pathways^12^.

Plasmonic nanomaterials and in particular gold nanoshells (GNSs)^13,14^ are potent hyperthermia mediators and can also be combined with anti-cancer drugs, imaging probes, and/or targeting ligands with the use of versatile Au–S immobilization strategies. GNSs have an adjustable plasmon absorption band that can be tuned in the red/near-infrared region^15^ where light has negligible interaction with physiological media (i.e. water, blood and tissue). In addition, they convert light to heat efficiently upon exposure to low energy laser irradiation. GNSs were first used as photothermal antennae in cancer therapeutics by West et al.^14,16^ reporting on the photothermal ablation of a breast tumor model and; since then, interesting studies have appeared utilizing GNSs in theranostics^17,18^, combinational therapeutics^19,20^, and bioimaging applications some of which have recently progressed to clinical trials^21^.

**Scheme 1:**
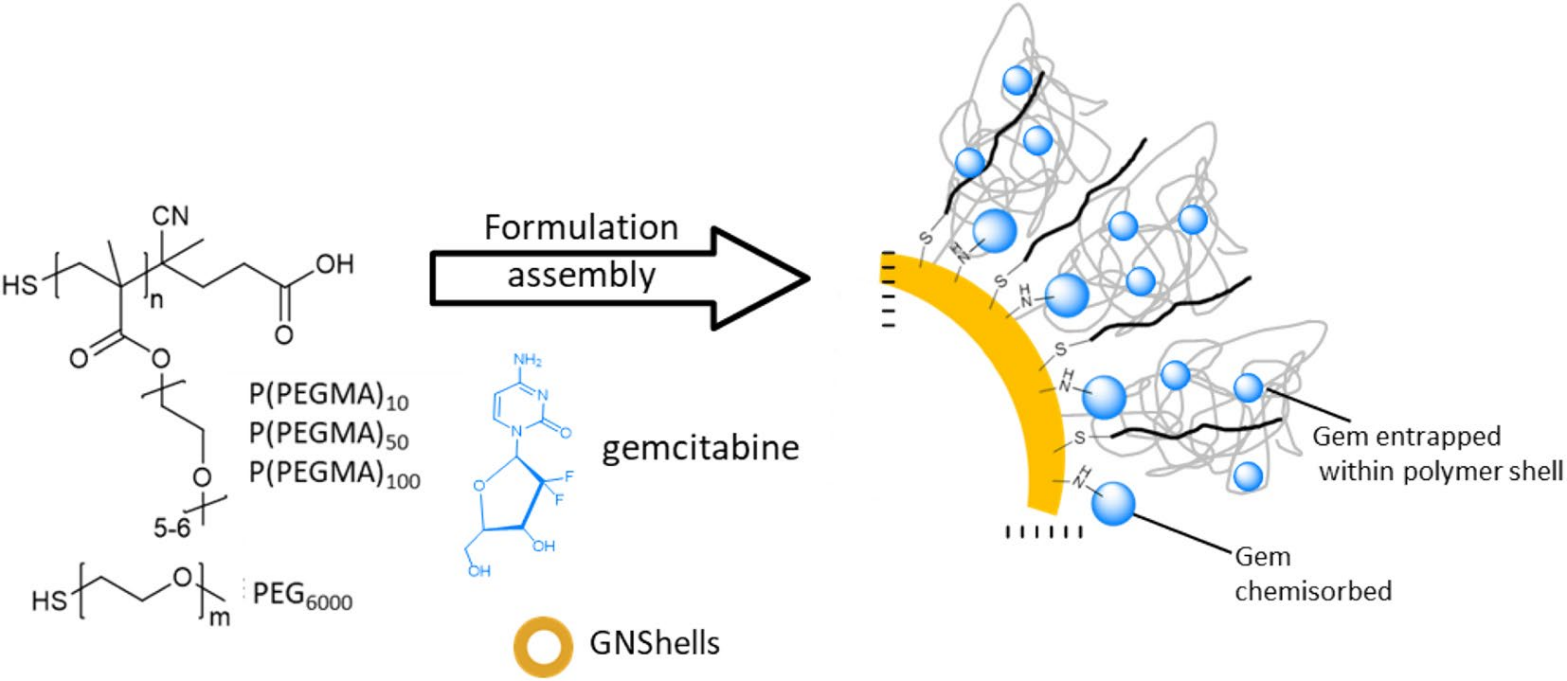
Proposed formulation strategy of PEG or P(PEGMA) coated GNShells co-loaded with gemcitabine.

The proposed formulation is simple and versatile in that we exploit the Au–N bond formation by GNSs and the primary amino group of GEM^22^ for direct drug loading and also the covalent immobilization of thiol-terminated PEG and PEG-type synthetic polymers of varying molecular weights for further steric protection and protein repulsion. We report the formulation of GEM loaded GNSs-coated by thiol-terminated poly(oligo(ethylene glycol) methacrylates) and we probe the roles of molecular weight and grafting density^23–25^, on the cellular uptake and ultimately the cytotoxic properties of the GEM-GNSs formulations against a pancreatic cell line (Scheme 1).

## Methods

### Materials

Dulbecco’s modified Eagle’s medium—high glucose (DMEM), Dulbecco’s phosphate buffered saline (DPBS), fetal bovine serum (FBS), glacial acetic acid, gold (III) chloride trihydrate (≥ 99.0%), hydroxylamine hydrochloride (≥ 98%), L-glutamine, phosphate buffered saline (pH 7.4), phosphate buffered saline (PBS) tablets, penicillin–streptomycin, silver nitrate (≥ 99.0%), sodium borohydride (99.99%), sodium-1-octane-sulphonate (≥ 99.0%), sodium acetate buffer solution (pH 5.2 ± 0.1), thiazolyl blue tetrazolium bromide (98%), and trisodium citrate dehydrate were all purchased from Sigma-Aldrich and used without further purification. Gemcitabine.HCl (Gem) was purchased from Sequoia Research Products Ltd. All solvents were of high-performance liquid chromatography grade and used without further purification. Distilled water (dH_2_O) was used for all the experiments. Silicon wafer (single side polished), <100>, P-type, with boron as dopant, diam. × thickness 3 in. × 0.5 mm was purchased from Aldrich. The human epithelia MiaPaCa-2 cell line was purchased from the American Type Culture Collection (ATCC). Poly(ethylene glycol) methyl ether thiol (average *M*_*n*_ = 6000, SH-PEG6000) was purchased from Sigma-Aldrich. Thiol-capped P(PEGMA) polymers of different molecular weights were synthesized in-house by reversible addition-fragmentation chain-transfer radical polymerization based on previously established protocols (Table S1)^26–29^.

### Preparation of materials

#### Synthesis of gold nanoshells

Hollow spherical GNShells were synthesized through the sacrificial oxidation of nanosilver templates by gold salt solution, following the protocol described by Prevo et al.^15^ with several modifications to prepare the nanoparticles at a larger scale. For the silver sols synthesis, 100 mL millipore water was added to a 250 mL round bottom flask. To this water, 0.2 mM silver nitrate (3.4 mg) and 0.5 mM sodium citrate (14.7 mg) were added while stirring. After about 5 min, 0.6 mL of a freshly prepared ice-cold solution of 100 mM sodium borohydride (6.3 mg mL^−1^) was injected quickly into the above mixture with vigorous stirring. The resulting silver colloids (yellow colour) were allowed to stir for a minimum of 2 h. The prepared silver seed particles were then grown to larger diameters by adding 1 mL of 200 mM hydroxylamine hydrochloride solution (13.9 mg mL^−1^). The colloidal suspension was subsequently stirred for 5 min. After further addition of 1.5 mL of 16.6 mM silver nitrate (2.83 mg mL^−1^), the silver nanocolloids were allowed to age overnight under magnetic stirring. The slightly turbid greenish-brown colored silver template was subjected to a galvanic replacement reaction with 0.25 mM tetrachloroauric acid at 60 °C. Dropwise addition of a 0.25 mM gold solution (1 mL) into the hot colloids of silver nanoparticles (60 °C) resulted in the complete disappearance of the silver plasmon band at 400 nm and appearance of broad absorption maximum at about 640 nm. The colour of the as synthesized GNShells was dark blue.

#### Surface functionalization and drug loading of the GNShells

The resulting GNShell suspension (1 mL) was centrifuged (Sigma 3–16 KL) at 8000 rpm for 20 min. The GNShell pellet was resuspended in 1 mL of dH_2_O and saturated with excess thiolated polymers.

The GNShells (1 mL) were mixed with each thiolated polymer (1:1 v/v) under magnetic stirring for 4 h at room temperature followed by centrifuging the PEGylated samples (8000 rpm, 20 min) using centrifugal Filter (Amicon Ultra-4 mL—with a membrane NMWL of 30 kDa) in order to remove the unbound polymers. Subsequently, the PEGylated GNShell pellets were re-suspended in 1 mL of Gem solution (0.1 mg mL^−1^) to prepare Gem-loaded GNShells and the mixture was allowed to stir at room temperature for 1 h. The purification of each sample was carried out by performing another centrifugation cycle (8000 rpm, 20 min) using centrifugal filter (Thermo Scientific Pierce concentrators (PES), 3 k MWCO, 0.5 mL). The resulting pellets were resuspended in dH_2_O (0.5 mL) for characterization and/or in PBS buffer (0.5 mL) for conducting in vitro cytotoxicity assays.

### Characterizations

#### Transmission electron microscopy (TEM)

The size and morphology of the GNShells were characterized by TEM on a FEI/PHILIPS CM120 BioTwin operating at 120 kV. After one cycle of centrifugation of GNShells, an aliquot of concentrated nanoparticle suspension was placed on a carbon coated copper grid. The excess of sample was drained gently by filter paper and allowed to dry out in open air for a few minutes. The grid was transferred to the sample holder and placed in the middle of the main microscope vacuum chamber to be introduced into the electron beam.

#### Dynamic light scattering and zeta potential

DLS measurements were carried using a Zetasizer Nano ZS, Malvern Instruments Ltd., UK, equipped with a HeNe 633 nm laser. The scattered laser beams were measured at the angle of 175° and the data were recorded by Malvern Zetasizer software 7.11. The samples were diluted in dH_2_O (1:1 v/v) and the measurements were conducted in plastic cuvettes at 25 °C.

The nanoparticles surface charge (zeta potential) was obtained using a Zetasizer Nano ZS, Malvern Instruments Ltd., UK. The samples were diluted in dH_2_O (1:1 v/v) and the measurements were conducted in disposable folded capillary zeta cells (Malvern) and performed 3 times, with 100 runs each time.

#### Thermogravimetric analysis (TGA)

TGA (TA instruments) was used to perform thermogravimetric analysis under nitrogen atmosphere. The concentrated colloids of the PEGylated GNShells were prepared by centrifuging 8 mL of PEGylated samples (8000 rpm, 20 min) using centrifugal filter (Amicon Ultra-4 mL—with a membrane NMWL of 30 kDa). The obtained concentrated PEGylated GNShells were placed in a platinum crucible and dried at room temperature prior to analysis. The temperature of sample was raised from 40 to 700 °C at a heating rate of 10 °C min^−1^ under an inert atmosphere of nitrogen (25 mL min^−1^). The amount of the polymer attachment to the surface of gold nanoparticles was found from the percentage mass loss over the temperature range of 300–450 °C, which was attributed to the decomposition of surface-bound polymer.

#### Raman spectroscopy

The Raman spectrum of the PEGylated GNShells was obtained to determine the binding interaction between thiol-capped polymers and the surface of the nanoparticles (Au–S bond). The PEGylated GNShell colloids were dried onto glass substrates and the films were characterized in a microRaman spectrometer (inVia Raman Microscope, Renishaw). For vibrational excitation, a diode laser (785 nm, Renishaw) with a maximum laser power of 300 mW was applied. Raman spectra in the range 150–950 cm^−1^ were collected using acquisition time of 60 s and 100% laser power per sample. The Raman spectrometer consists of a Renishaw spectrograph system based on the use of Kayser notch filters with a sensitive CCD detector coupled to a microscope for point-by-point analyses.

#### X-ray photoelectron spectroscopy (XPS)

XPS spectra were collected on a Thermo K-alpha instrument utilizing a 72 W monochromated Al-Kα X-ray source (with photon energy of 1486.6 eV). The depth profiles were completed using an Ar^+^ ion gun at 3000 kV. Samples were prepared by drop coating of nanoformulations on a clean silicon wafer, and the drops were allowed to air dry. The nano-formulations coated silicon wafers mounted onto the XPS specimen holder with vacuum compatible, double-sided adhesive carbon tape before the measurement. Clean gloves and tweezers were used to avoid contaminating the wafer. Binding energies were referenced against the Au 4f 7/2 at 83.58 eV and the spectra were analyzed using CasaXPS version 2.3.16.

#### High performance liquid chromatography (HPLC) analysis for the quantification of the drug loading efficiency

The HPLC method for the quantification of Gem was developed using an Agilent Technologies 1200 Series HPLC system. The data was acquired and analyzed using ChemStation for LC software, also by Agilent Technologies, UK. The chromatographic separation was achieved using a Phenomenex Synergi 4 μm Polar-RP 80 Å, LC Column 250 × 4.6 mm.

The surface-functionalized GNShells (GNShells + thiol-end polymer + Gem) were centrifuged at 8000 rpm for 20 min at 25 °C using a centrifugal concentrator (Thermo Scientific Pierce concentrators (PES), 3 k MWCO, 0.5 mL). The obtained 500 μL supernatant was diluted up to 800 μL with HPLC grade water followed by addition of 200 μL methanol to adjust the total volume at 1 mL.

The prepared HPLC samples were isocratically eluted with a mobile phase of buffer and acetonitrile (95:5 v/v). The aqueous buffer was prepared by dissolving 3.86 g of ammonium acetate (0.05 M) and 1 g of sodium-1-octane-sulphate in 1 L of HPLC grade water and adjusted to pH 4 with glacial acetic acid.

The mobile phase was pumped through the column at a flow rate of 1 mL min^−1^. The UV detector was set at 270 nm and the injection volume was 20 μL.

The Gem standard solutions used for quantification were prepared by suitably diluting 75 μg mL^−1^ working standard of the drug in a mixture of 80:20 water:methanol. The duration of each run was 20 min and the Gem peak appeared at around 8.5 min. The measurements were performed in triplicate and the Gem loading efficiency (LE) was obtained using the following equation

1 $$LE \left(\%\right)= \frac{{Gem}_{Original }- {Gem}_{Supernatant}}{{Gem}_{Original}}$$

### Drug release study

10 mL of each Gem-loaded GNShells samples (GNShells + thiol-end polymer + Gem) were centrifuged (8000 rpm, 20 min) using centrifugal filter (Amicon Ultra-15 mL—with a membrane NMWL of 3 kDa). The obtained concentrated nanoformulation was resuspended in 10 mL release medium at different physiological pH level: sodium acetate buffer solution (pH 5.2 ± 0.1), phosphate buffered saline (pH 7.4). The temperature and stirring of the system were maintained at 37 °C and at 100 rpm, respectively. 500 μL of sample was withdrawn at predetermined time points for 48 h (*n* = 3). After each sample collection, the same amount of fresh media was added back to the release medium to maintain the sink conditions. The collected samples were centrifuged; using centrifugal filter (Thermo Scientific Pierce concentrators (PES), 3 k MWCO, 0.5 mL) for 20 min at 8000 rpm and then the samples’ supernatant were subjected to HPLC analysis. The results were presented in terms of cumulative release as a function of time (mean ± SD). The cumulative percentage drug released versus time was calculated using the following equation:

2 $$Cumulative \, drug \,release \left(\%\right)= \frac{{C}_{t}}{{C}_{\infty }} \times 100$$

where, C_t_ is the mass of drug released at time t and C_∞_ refers to the initial loaded mass.

### Photothermal studies

#### Determination of the photothermal conversion efficiency

The laser-induced temperature elevation of GNShells, GNShells + SH-P(PEGMA)100 and GNShells + SH-P(PEGMA)100 + Gem was investigated by irradiating 1 mL of each colloidal suspension (OD = 1) (Table S3) in quartz cells (surface area = 1 cm^2^) with a continuous-wave fiber coupled diode laser with a center wavelength of 640 nm and output power of 0.9 W cm^−2^ (540 J 600 s cm^−2^) for 10 min. Water, PBS (pH 7.4), sodium acetate buffer solution (pH 5.2) and cell culture medium were used as control groups. The temperature was monitored every 1 min over 20 min, including 10 min cooling time period, by a digital thermometer (Digitron TM22) with a thermocouple probe submerged in the colloidal suspensions in the quartz cuvettes. The photothermal conversion efficiency (η) of the samples was calculated based on a protocol described by Pinchuk et al.^30^.

#### Determination of the photothermal effect on drug release profiles

Pellets obtained from centrifugation of 10 mL GNShells + SH-P(PEGMA)100 + Gem, were resuspended in 10 mL of release medium at different physiological pH levels: sodium acetate buffer solution (pH 5.2 ± 0.1), and PBS (pH 7.4). All the release studies were carried out at 37 °C and stirring was maintained at 100 rpm. The release behavior of Gem from GNShells + SH-P(PEGMA)100 + Gem with and without laser irradiation was monitored. One hour after starting the procedure, stirring samples (10 mL) in glass vials (surface area = 4 cm^−2^) were irradiated with red laser (640 nm, 0.9 W cm^−2^/1080 J 1200 s cm^−2^) for 20 min. 0.5 mL of each sample was withdrawn at fixed time intervals and the same amount of fresh media was added back to the release medium to maintain sink conditions. The collected samples were centrifuged using centrifugal filter (Thermo Scientific Pierce concentrators (PES), 3 k MWCO, 0.5 mL) for 20 min at 8000 rpm and then, the samples supernantant were collected. By comparing to the concentration of the pure Gem (equivalent to 100% Gem release) in each buffer, the concentration of released Gem (from the collected supernatants) was quantified by HPLC analysis. The results were presented in terms of cumulative release as a function of time. The cumulative percentage drug released versus time was calculated using Eq. 2.

### In vitro assays

#### In vitro cytotoxicity studies

The experimental protocols used were based on our previous works^31,32^. MiaPaCa-2 cells (ATCC) cells are large with abundant cytoplasm, exhibit a high degree of aneuploidy, have a tendency to grow on the top of other cells, eventually growing free in suspension (ATCC). The cells were incubated in DMEM supplemented with 10% fetal bovine serum, 1% penicillin–streptomycin and 1% L-glutamine. MiaPaCa-2 cells were seeded in a 96-well plate at a density of 1 × 10^4^ cells per well and incubated in humidified atmosphere with 5% CO_2_ for 24 h before the assay. The culture medium containing different concentrations of drug ranging from 0.001 to 100 μmol L^−1^ was added to the adherent cells while control cells remained untreated.

After incubation for 48 and 72 h with different concentrations of drug, the medium was replaced by 100 μL of fresh medium and 25 μL of MTT stock solution (5 mg mL^−1^ in PBS) and incubated for an additional 4 h. Subsequently, the medium was gently removed and the water insoluble formazan crystals were dissolved in 200 μL of DMSO. The plates were shaken for 2 min at room temperature before measuring the optical density (OD) at 570 nm on a SpectraMax M2/M2e Multimode Microplate Reader, with SoftMax Pro Software. The obtained data was analyzed with the help of Prism software to acquire half maximal effective concentration (EC_50_) value. The same procedure was repeated for treating the cells with formulation (GNShells + thiol-capped polymer + Gem) at concentration of 0.001–100 μmol L^−1^ Gem equivalent doses. All the MTT experiments were performed in triplicate and the variation in the readings were shown as error bars (± SD).

#### In vitro photothermal studies

MiaPaCa-2 cells were incubated in Dulbecco’s modified Eagle’s medium–high glucose supplemented with 10% fetal bovine serum, 1% penicillin–streptomycin and 1% L-glutamine. The cells were then seeded in a 96-well plate at a density of 1 × 10^4^ cells per well and incubated at 37 °C in humidified atmosphere with 5% CO_2_ for 24 h before addition of free Gem, GNShells + SH-P(PEGMA)100 and GNShells + SH-P(PEGMA)100 + Gem. Three batches of culture medium containing free Gem and GNShells + SH-P(PEGMA)100 and GNShells + SH-P(PEGMA)100 + Gem at different concentrations (or equivalent concentrations) of Gem, ranging from 0.001 to 100 μmol L^−1^ were added to the adherent cells while control cells remained untreated. After incubation for 24 h, treated/untreated cells were irradiated with red laser (640 nm, 0.9 W cm^−2^) at various exposure times (1, 5 and 10 min). For chemotherapy treatment alone, the cells were not exposed to laser light (Table S2). After laser exposure, the cells were maintained at 37 °C under 5% CO_2_ for additional 24 h and 48 h. Finally, the MTT assay was utilized to study the cytotoxicity of drug/nanoformulation after predetermined times (Scheme S1). The obtained data was analyzed with the help of Prism software to acquire the half maximal effective concentration (EC_50_) value.

#### Chemo-photo-thermal synergism studies

MiaPaCa-2 cells were seeded onto 6-well plates (surface area of each well = 9.5 cm^−2^) at a density of 1 × 10^6^ cells per well and incubated at 37 °C in humidified atmosphere with 5% CO_2_. On the next day, the medium was replaced with fresh medium containing 100 μmol L^−1^ of free Gem, GNShells + SH-P(PEGMA)100 + Gem (Gem equivalent dose) and GNShells + SH-P(PEGMA)100 (GNShells + SH-P(PEGMA)100 + Gem equivalent dose). After 24 h of incubation, the cells were irradiated with red laser (640 nm) at a power level of 0.9 W cm^−2^ for 60 min (3240 J 3600 s cm^−2^). After further 48 h of incubation, the cells were harvested with trypsin and the populations of survived cells after each treatment were counted. The population of cells after treatment with GNShells + SH-P(PEGMA)100 + Gem + laser was compared with the number of cells after an additive interaction (P _Additive_) in order to determine the type of interaction between the applied nanoformulation and the photothermal treatment (synergistic, antagonistic, or additive).

*P *_*Additive*_ was calculated using the following equation:

3 $${P}_{Additive}=\left({f}_{chemotherapy} \times {f}_{phototherapy}\right){ P}_{0}$$

where, *P *_*additive*_ represents the number of cells after an additive interaction, *f*_*chemotherapy*_ is the fraction of survived cells after treatment with Gem + laser exposure, *f*_*phototherapy*_ is the fraction of survived cells after treatment with GNShells + SH-P(PEGMA)100 + Gem + laser exposure, and *P*_*0*_ represents the starting population of cells.

#### Cellular uptake studies

MiaPaCa-2 cells were seeded in 6-well plates at a density of 1 × 10^5^ cells per well (surface area of each well = 9.5 cm^−2^) and incubated at 37 °C in humidified atmosphere with 5% CO_2_ until they reached 70–80% confluency. The medium was replaced with fresh medium containing 100 μL of GNShells + SH-P(PEGMA)100 (~ 1 × 10^9^ particles per mL) followed by irradiating the cells with red laser (640 nm) at a power level of 0.9 W cm^−2^ for 60 min (3240 J 3600 s cm^−2^) while control cells remained untreated (without photothermal treatment). After 18 h of incubation, the cells were rinsed three times with PBS and the cell pellets were collected by trypsinization and centrifugation. The cell pellets were fixed in 0.1 M cacodylate buffer (pH 7.3) containing 2.5% glutaraldehyde for 2 h at 4 °C, and post-fixed with 1% osmium tetroxide in 0.1 M cacodylate buffer (pH 7.3) for 1 h at 4 °C. The cells were then dehydrated in a graded series of ethanol solutions (30% for 15 min, 50% for 15 min, 70% for 15 min, 90% for 15 min, and 100% for 15 min twice) and embedded in Epon resin. Ultrathin sections of embedded cells were cut and transferred on to copper grid, stained with lead citrate, and observed in a Philips BioTwin CM120 (FEI) transmission electron microscope, operating at 120 kV.

#### Inductively coupled plasma mass spectrometry (ICP-MS)

MiaPaCa-2 cells were seeded in 6-well plates at a density of 1 × 10^5^ cells per well and incubated at 37 °C in humidified atmosphere with 5% CO_2_ until reaching 70–80% confluency. The medium was replaced by fresh medium containing 20 μL PEGylated GNShells followed by irradiating the cells with red laser (640 nm) at a power level of 0.9 W cm^−2^ for 30 min while control cells remained untreated (without phothothermal treatment). After 12 h of incubation, the medium was removed and the cells were rinsed three times with phosphate buffer saline (PBS) and cell pellets were collected using trypsin. The cells were counted and then centrifuged at 1000 rpm for 10 min. Supernatants were removed, and the pellets were digested with 500 μL of fresh aqua regia for 30 min and then diluted to a total volume of 10 mL with 5% aqua regia. Samples were analyzed on a Bruker M90 ICP-MS equipped with an autosampler. A series of gold standard solutions (2, 1 and 0 ppb) were prepared in 5% aqua regia and the resulting calibration curve was used to determine the gold amount taken up by the cells in each sample (ppb).

#### Clonogenic cell survival assay

The assay was developed based on our previous protocols^31^. 1 × 10^5^ MiaPaCa-2 cells were seeded in 6-well plates (using 2 mL of culture medium). Once the cells reached 70–80% confluency, each well (surface area of each well = 9.5 cm^−2^) was treated with the different concentration of Gem (ranging from 0.001 to 100 μmol L^−1^), GNShells + SH-P(PEGMA)100 + Gem (Gem equivalent doses) and GNShells + SH-P(PEGMA)100 (GNShells + SH-P(PEGMA)100 + Gem equivalent doses). One well was remained untreated as a control. After 24 h, the treated and untreated wells were irradiated with red laser (640 nm) at a power level of 0.9 W cm^−2^ for 60 min (3240 J 3600 s cm^−2^). For chemotherapy alone, the cells were not exposed to laser light. The cells were harvested by trypsinization, counted and subsequently specific number of cells (100 cells) from each well was seeded in a new 6-well plate (using 2 mL of culture medium) in triplicate. After 14 days, the colonies were washed with PBS and then fixed using methanol and acetic acid in ratio 3:1. The colonies were stained with 0.5% crystal violet solution (diluted with methanol) for 5 min. The stained plates were rinsed in the tray full of distilled water and left in the fume hood overnight to dry. Colonies appeared as clusters of violet stained cells and could be visualized with the naked eye. The number of air-dried colonies for the average of three colony counts for each plate was recorded. A cluster of 50 or more cells were counted as one colony. After counting colonies, plating efficiency (PE) was calculated by dividing the number of colonies counted by the number of cells plated and then multiplying by 100:

4 $$Plating\,efficiency \left(PE\right)= \frac{No.\,of\,colonies\,counted}{No.\,of\,cells\,plated} \times 100$$

PE was determined to investigate the percentage of the single cells seeded in the plates that form a colony. PE of the control was considered as 100%. By determining PE, Survival fraction of the single cells seeded in the plates also calculated by dividing the PE of the treated cells by the PE of the control and then multiplying by 100:

5 $$Survival\,fractions \left(SF\right)= \frac{PE\,of\,the\,treated\,cells}{PE\,of\,the\,control} \times 100$$

The survival fraction was calculated to determine the fraction of surviving cells after the exposure to the different concentration of drug and laser exposure.

## Results and discussion

### Gold nanoshells

**Figure 1:**
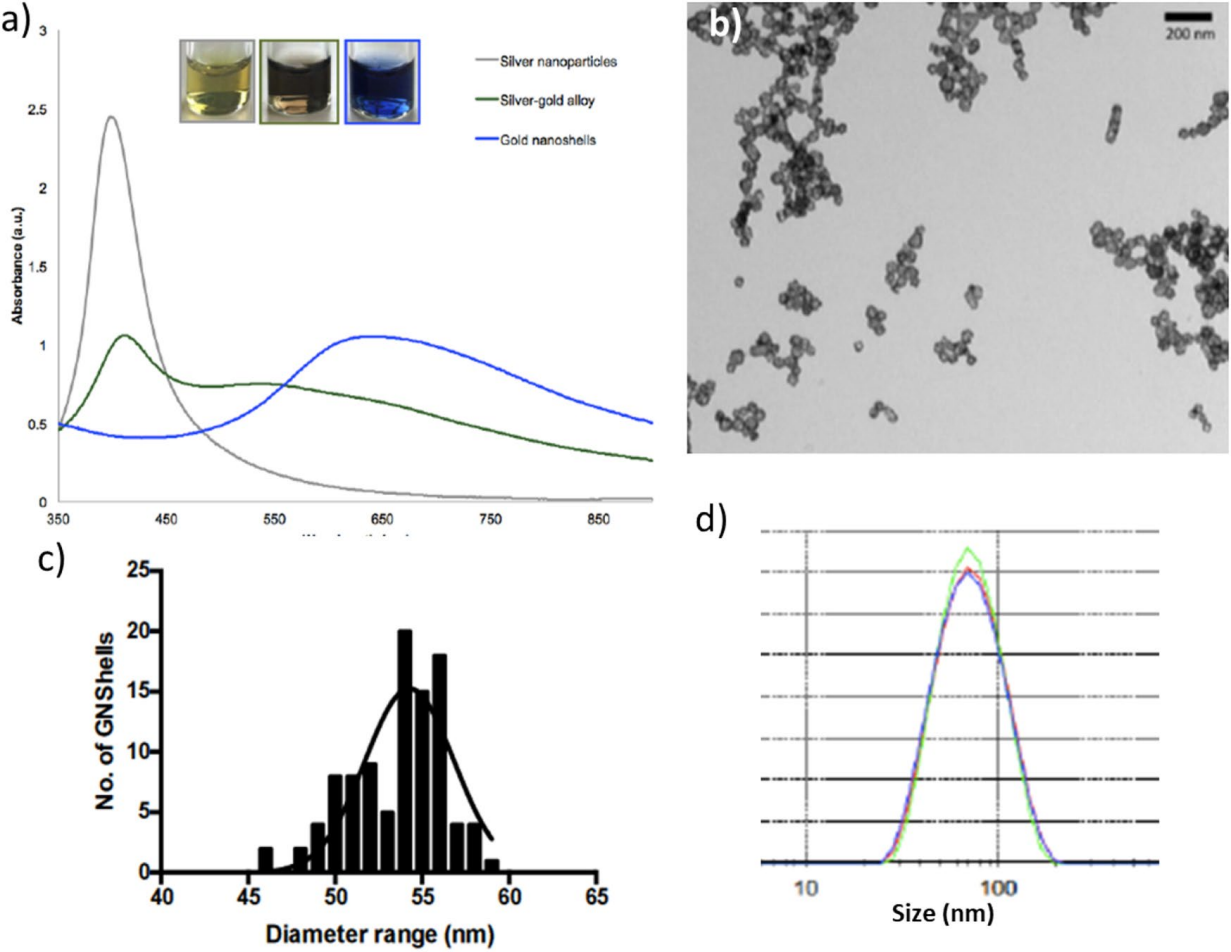
(**a**) UV/Vis absorption spectra of the silver colloids (grey) and the silver-gold alloy bimetallic nanoparticles (dark green) and the originally synthesized GNShells (blue), (**b**) TEM image of the as synthesized GNShells, (**c**) particle size distribution from TEM images and (**d**) DLS hydrodynamic size distribution of GNShells.

The hollow spherical GNShells were synthesized based on the galvanic replacement reaction method with gold ions as precursors and silver nanoparticles as sacrificial templates. The surface plasmon resonance (SPR) of the GNShells was tuned to red/NIR wavelengths by controlling the dimension of interior cavity and shell thickness. The GNShells have an SPR maximum value at 640 nm, which is situated in the red/NIR optical window where photons penetrate deeper into living tissue^33–35^. The disappearance of the absorption peak at about 400 nm confirmed the oxidation of silver atoms during the transmetallation reaction and deposition of the gold in the form of a shell around the silver core (Fig. 1a).

The morphology of the synthesized GNShells was evaluated by TEM; the GNSHells were hollow, mostly spherical and fairly uniform in size (Fig. 1b). The size distribution of 100 GNShells was measured from several TEM images and a frequency histogram of diameter range was constructed (Fig. 1c). The frequency distribution curve of the GNShells had Gaussian size distribution. The most frequent GNShells size (mode) that appeared in the data set was 54 nm and the average effective diameter was measured to be 53.5 ± 2 nm. The hydrodynamic diameter (D_h_) of the as prepared GNShells was also measured by DLS and the mean D_h_ was found to be 60.44 ± 12 nm slightly higher than the TEM results due to the hydrodynamic shell of the GNSHells in suspension (Fig. 1d).

### GNSHells functionalization

Initially, we examined the morphology of the polymer coated GNShells with TEM. It was possible to trace a thin organic layer in all polymer-coated batches showing the successful coating procedure (Figure S1).

**Figure 2:**
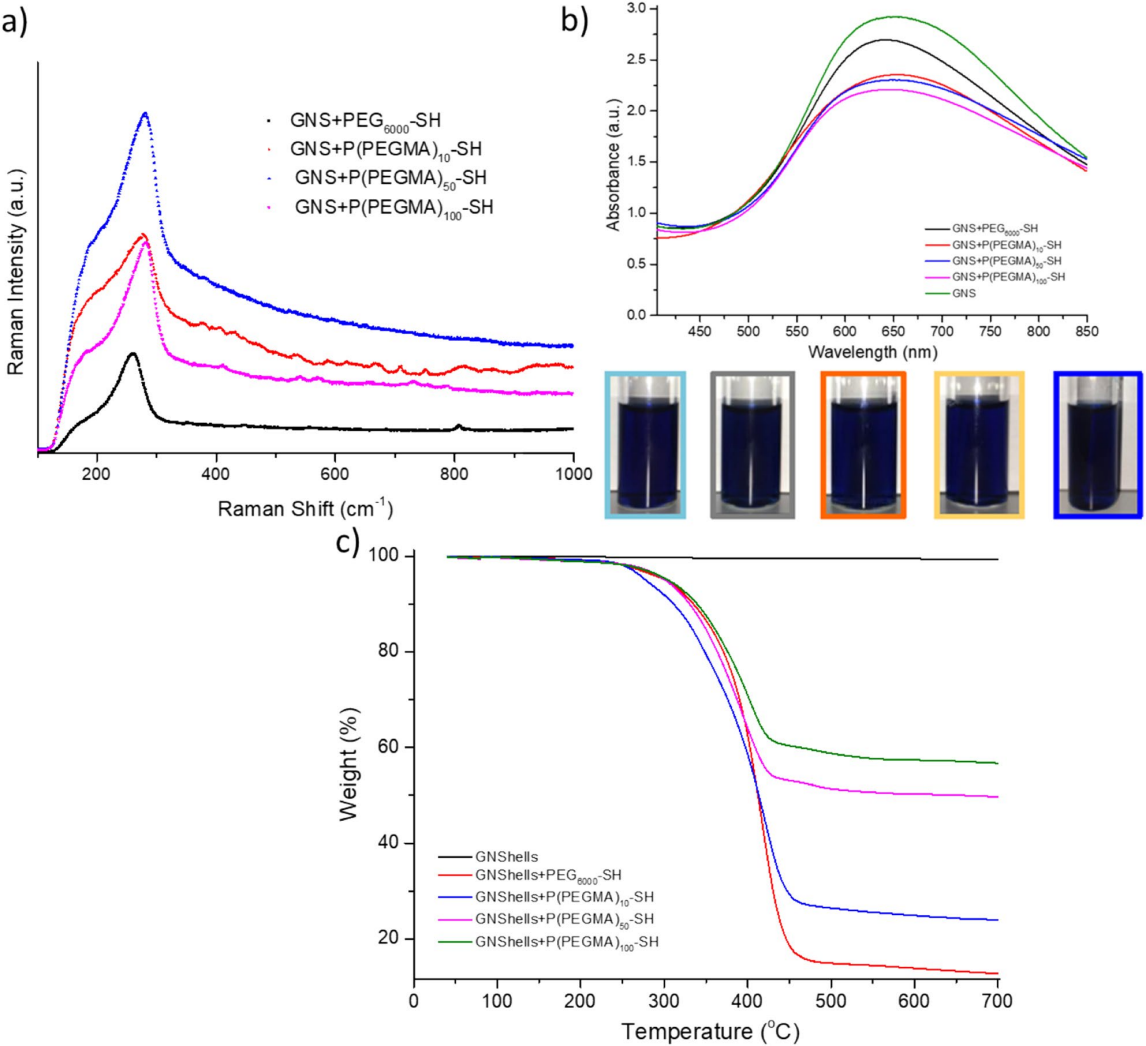
(**a**) Raman spectra of GNSHells + SH-P(PEGMA)_10_, GNSHells + SH-P(PEGMA)_50_, GNSHells + SH-P(PEGMA)_100_, GNSHells + SH-PEG_6000_, (**b**) UV/Vis spectra of polymer coated GNSHells and (**b**) their optical appearance in suspension; coated with PEG6000 (light blue), P(PEGMA)10 (grey), P(PEGMA)50 (orange), P(PEGMA)100 (yellow), and PEG6000 (blue) and c) thermogravimetric analysis polymer coated GNSHells compared with a non-coated sample.

We next studied the attachment of the polymers and gemcitabine on the surface of GNSHells with Raman spectroscopy. Gold (I) with its [Xe] 4f^14^5d^10^ electronic configuration is a soft metal ion and therefore according to the Pearson acid base concept has a preference for soft donor ligands such as sulfur^36^. The thiol group is the most commonly selected anchor group for adsorption onto the surface of the gold nanoparticles because of the strong chemical bond, with high bond enthalpy of 418 kJ mol^−1^, between gold and sulfur that makes this interaction desirable as a robust attachment mechanism^37^. Initially the Au–S interaction was assessed by Raman spectroscopy (Fig. 2a); all Raman spectra were recorded from the coffee ring area, where PEGylated GNShells were closely packed, to ensure precise SERS detection of the grafted polymers. Characteristic Raman peaks were observed between 267–285 cm^−1^ that correspond to the vibrational modes of SH-PEG/PEGMA molecules adsorbed on the GNShells’ surface. The stretching modes of Au–S are typically reported to be between 200–240 cm^−1^. However, this mode may shift towards higher wavenumber (300–310 cm^−1^) when the sulfur atom is attached to longer polymer chains^38–40^. The results confirmed the covalent attachment of the SH-PEG/PEGMA molecules to the GNShells core after displacing the citrate stabilizer owing to the stronger binding affinity of the sulfur atom. Polymer grafting resulted in a slight red-shifting accompanied by peak broadening and a decrease in the intensity (Fig. 2b). This set of data implies that PEGylation partially reduced the inter-particle distance between the neighboring particles and induced particle aggregate assemblies which in turn shifted the SPR band to lower energies, corresponding to longer wavelengths of the spectrum. These red-shifting and SPR damping are the consequence of the partial increase of the relative contribution of surface plasmon scattering to the total light extinction for larger size particles and is in good agreement with Mie theory. The band broadening is due to unavoidable coupling of the plasmon resonance from multiple GNShells and contribution of higher order electron oscillation or the altering of the local refractive index around the GNShells as the result of PEGylation^41^. Expectedly all formulations retained a broad absorption band from 600–800 nm which is suitable for photothermal therapeutics activated by red or near-infrared laser sources which allows for deep tissue penetration and renders the formulations clinically relevant.

Next, TGA was performed to evaluate the amount of grafted polymers associated with the gold surface of the GNShells (Fig. 2c). As expected, no weight loss step was observed in the TGA trace of the pristine GNShells indicative of their purity and absence of organic material. For all polymer-coated samples, about 2% weight loss around 100 °C was due to the loss of adsorbed water. Up to 5% weight loss before 300 °C could be assigned to the presence of grafted minor impurities possibly by the polymer samples. The major weight reduction occurring between 300 and 450 °C was attributed to the thermal degradation of the P(PEGMA) and PEG polymers. The mass loss (%) from the TGA thermograms was found to be 80% for PEG6000, and 65.50%, 43.70% and 36.70% for P(PEGMA)10, P(PEGMA)50 and P(PEGMA)100, respectively. Of note is the high association of PEG6000 with GNShells and then a pattern of increasing polymer association inversely associated with increasing the molecular weight of the P(PEGMA) polymers. Overall, the values we obtain are relatively high compared to PEG coated spherical gold nanoparticles^42^ but in line with other studies of ligand-coated GNSs^43^. These findings are attributed to two factors: 1. The actual Au–S chemisorption on the gold surface which is possible to take place in both the inner and outer surface of the GNSs as we confirmed with Raman spectroscopy and XPS (for polymer coated GNShells + Gem, Table S4 and Figure S2) and 2. The possible physisorption of additional polymer chains in the form of physical entanglements resulting in additional organic material confined on the GNSs which is more pronounced with higher molecular weight P(PEGMA) homopolymers as demonstrated by the TGA data and the TEM images (Figure S1)^44^. The DLS results after polymer functionalization followed a similar trend: a high increase of the hydrodynamic radius by PEG6000 functionalization and then an increasing trend of the radius by increasing the molecular weight of P(PEGMA) (Table 1). The colloidal stability was monitored for 14 days which confirmed the longer term stability as the hydrodynamic size and the polydispersity index of the samples remained unchanged during this timeframe (Figure S3).

**Table 1:** Hydrodynamic size (D_h_) of the GNShells after PEGylation with thiol-terminated polymers.

D_h_ (nm)			
GNShells + SH-PEG6000	GNShells + SH-P(PEGMA)10	GNShells + SH-P(PEGMA)50	GNShells + SH-P(PEGMA)100
70.63 ± 1.91	64.94 ± 2.42	67.87 ± 2.20	70.58 ± 1.46

Finally, the zeta potential of the polymer coated samples was also evaluated and monitored over a period of 14 days. The ζ-potential value for the uncoated citrate stabilized GNShells was − 37.5 mV and increased to − 21.6 mV, − 34 mV, − 30.5 mV and − 26.3 mV for PEG6000, P(PEGMA)10, P(PEGMA)50 and P(PEGMA)100 grafted GNShells, respectively (Figure S4). The batch of GNShells coated with mPEG6000-SH had lower ζ value compared to that of the P(PEGMA) polymers with a carboxylic acid functional group on each polymer chain end. Higher molecular weight polymers yielded higher levels of neutralization given the less charge per mass of polymer chain. Hence the GNSHells adopted steric repulsive properties owing to the ethylene glycol-based shell and were found to be colloidally stable for 14 days without any noticeable difference as previously mentioned.

### Photothermal properties of GNShells

Before testing the photothermal effects of GNShells on cells, we studied their photothermal properties in PBS and sodium acetate buffer (Figure S5). As expected, all GNShell batches induced sharp temperature increase within 10 min of irradiation (λ = 640 nm, 0.9 W cm^−2^) of a *Δ*T of more than 20 °C compared to a mere *Δ*Τ of ca. 10–11 °C that was recorded with the GNShell-free buffer media controls. From preliminary cytotoxicity experiments, we shortlisted P(PEGMA)100 coated samples as they performed better in in vitro studies and we observed that polymer coating had no effect in the heating capacity of the GNShells (Figure S6a). From the heating and cooling experimental dataset (Figure S6b) it was possible to calculate the photothermal conversion (η) performance of the GNShells with the methodology used by Roper et al.^45^ which was found to be 21% and is in accord with previous studies from the literature^46–48^.

### Drug loading and release

The drug loading efficiency of Gem onto the polymer coated GNSHells was assessed by HPLC. A loading efficiency of Gem of 59% was achieved with the PEG6000 coated samples, which was about 5–6% lower than the P(PEGMA) coated samples (65%, 64% and 65%, for P(PEGMA)10, P(PEGMA)50 and P(PEGMA)100, respectively). As the prepared GNShells were homogeneous in size, this small loading efficiency (%) variation could be related to the structural difference between the commercial PEG and the synthesized P(PEGMA) polymers. Although the bulky and relatively rigid structure of the thiol-terminated P(PEGMA) polymer chains can reduce the drug access to the binding sites i.e. they can limit the conjugation of Gem molecules to the GNShells’ surface by inhibiting the direct Au–N bond formation, Gem molecules can be trapped in inter-polymer complexes which can be formed by the grafted PEGMA chain. Therefore, it is likely that a fraction of the drug is trapped within polymer chain entanglements and another is chemisorbed on the surface of GNShells and this is more pronounced in the P(PEGMA) coated samples. This scenario is in accord with the reported XPS composition-depth profiles (atomic percent against etch time (s)) of Gem-loaded GNShells (Table S4, and Figure S2). It is worth mentioning that increasing the initial drug/GNShells ratio did not improve the loading efficiency (%), which indicates some sort of saturation binding that was reached for the concentrations studied. Therefore, with the obtained optimal loading efficiency, further characterization of the Gem release profile was carried out.

**Figure 3:**
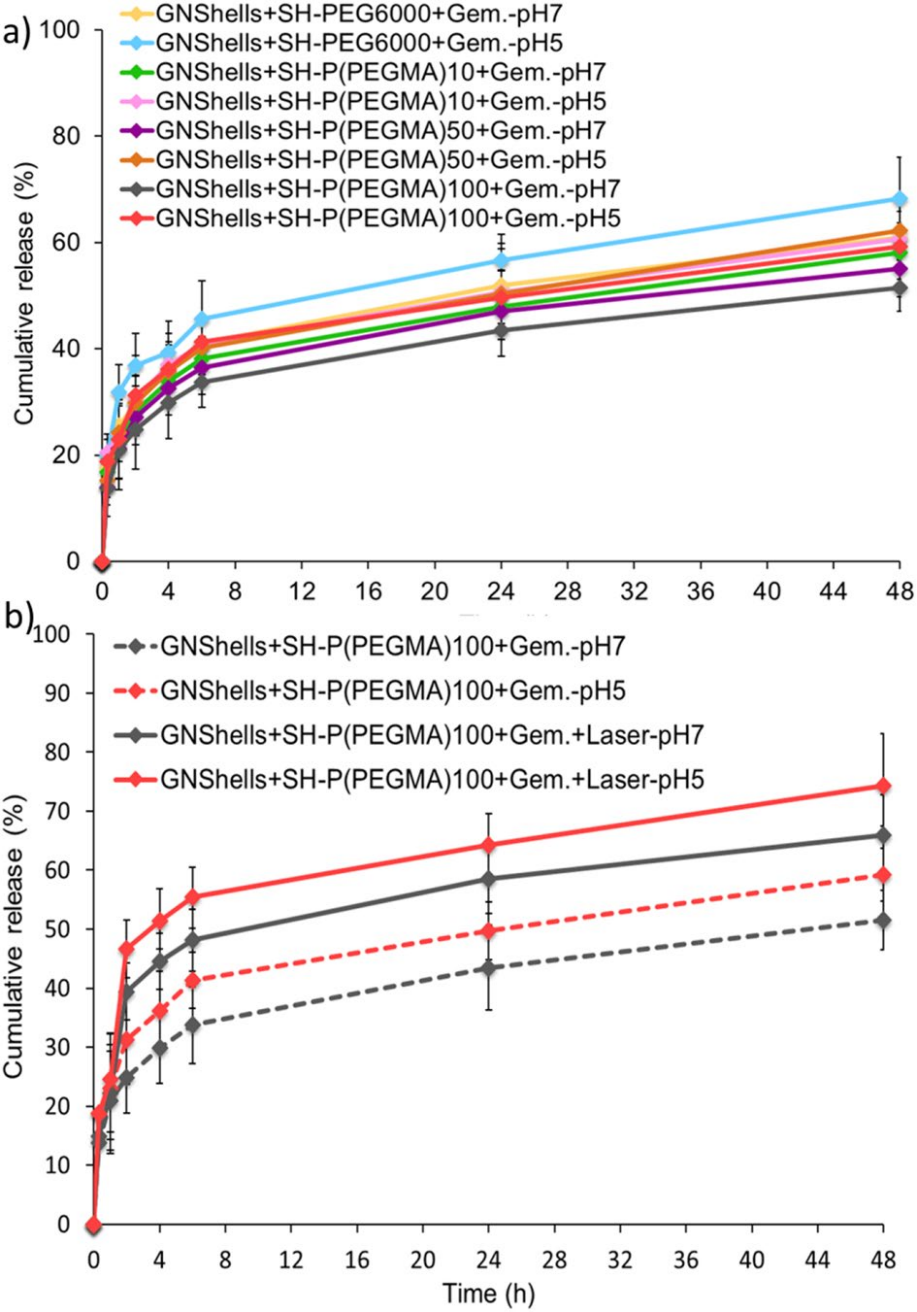
(**a**) Gem release profile from polymer coated GNShells for 48 h at different pH and (**b**) the effect of laser irradiation on the GM loaded GNSHells for the total release timeframe of 48 h. The data represented as the mean of three experiments.

The drug release behavior of the Gem-loaded nanoshells at 37 °C was investigated in phosphate buffer saline (pH 7.4) to mimic the physiological environment and was also performed in acetate buffer saline (pH 5.2) to mimic the acidic late endosome intracellularly. The drug release profile (Fig. 3a) in both pH conditions showed two stages, a relatively rapid release of Gem within 6 h followed by a constant release of lower rate. The initial rapid release, known as “burst effect”, occurs probably due to the physically adsorbed drug on the outer shell of the polymer layer as previously mentioned^49^. The slower drug release after 6 h could be due to the diffusion barrier created by the PEG/P(PEGMA) coating. The PEG chains interact with one another so that a complex could form amongst the grafted chains which in turn delays the release from the P(PEGMA) coated samples compared to the PEG coated counterparts. Furthermore, the complexation effects of the grafted polymer conformations provide an additional diffusion barrier for a more tortuous path for drug release^50^ as the P(PEGMA)100 coated GNShells showed the slower release rate compared to all other nanoformulations. The percentage of cumulative release in PBS at pH 7.4 was found to be 41% at 6 h and 61% at 48 h from the PEG6000 coated GNShells. Moreover, in PBS (pH 7.4) at 6 h, 38%, 36% and 33% of Gem was released from P(PEGMA)10, P(PEGMA)50, and P(PEGMA)100 coated GNShells, respectively, while the release at 48 h was 58% for P(PEGMA)10, 55% P(PEGMA)50 and 51% for P(PEGMA)100 coated GNShells. In the acidic media, at 6 h, 45%, 41%, 40% and 41% Gem was released from PEG6000, P(PEGMA)10, P(PEGMA)50 and P(PEGMA)100 coated samples, respectively. At 48 h the cumulative release values were 68% for PEG6000, 60% for P(PEGMA)10, 62% for P(PEGMA)50 and 59% for P(PEGMA)100 coated samples. The slightly enhanced release profiles in the acidic media by 4–8%, though statistically not significant, are attributed to the enhanced solubility of Gem due to the protonation of the primary amine group under acidic conditions^51^.

Irradiation of the samples with the CW laser resulted in higher drug release rates (Fig. 3b); for example, irradiation of the P(PEGMA)100 coated samples with the laser for 20 min (0.9 W) resulted in 39.4% and 46.4% release of GEM in pH 7.4 and 5.2, respectively. The total release of GEM from the irradiated formulation reached 65.9% and 74.3% at pH 7.4 and 5.2, respectively. This increment is attributed to the promoted dissociative interactions between GEM and gold which in turn accelerate the overall rate of release and also to the increased diffusion rate due to the elevated temperature of the medium of the irradiated samples.

### In vitro studies

The EC_50_ values for all Gem-loaded samples were determined against MiaPaCa-2 cells. It was found that all samples had inferior EC_50_ compared to the parent drug by one order of magnitude (Table 2).

**Table 2:**
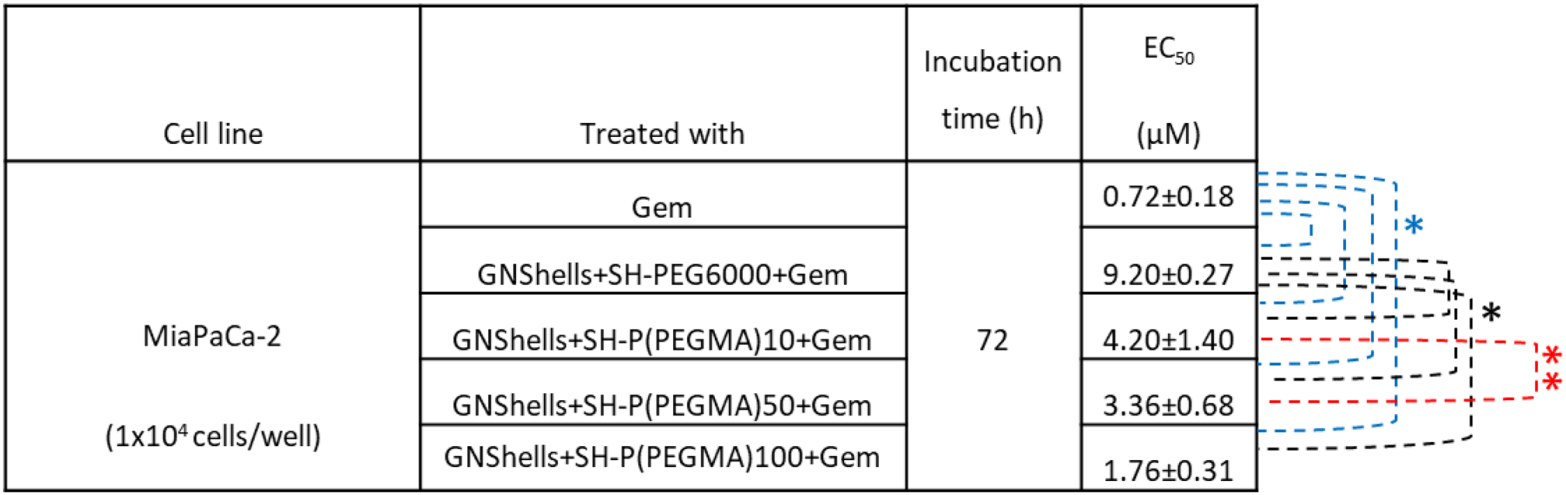
EC_50_ values of native Gem vs GNShells loaded by MTT assay on MiaPaCa-2 cells (triplicate experiments); * p < 0.01 denotes statistical significance (blue and black sample comparisons), and ** denotes statistically insignificant values, p > 0.01 (samples compared in red).

**Figure 4:**
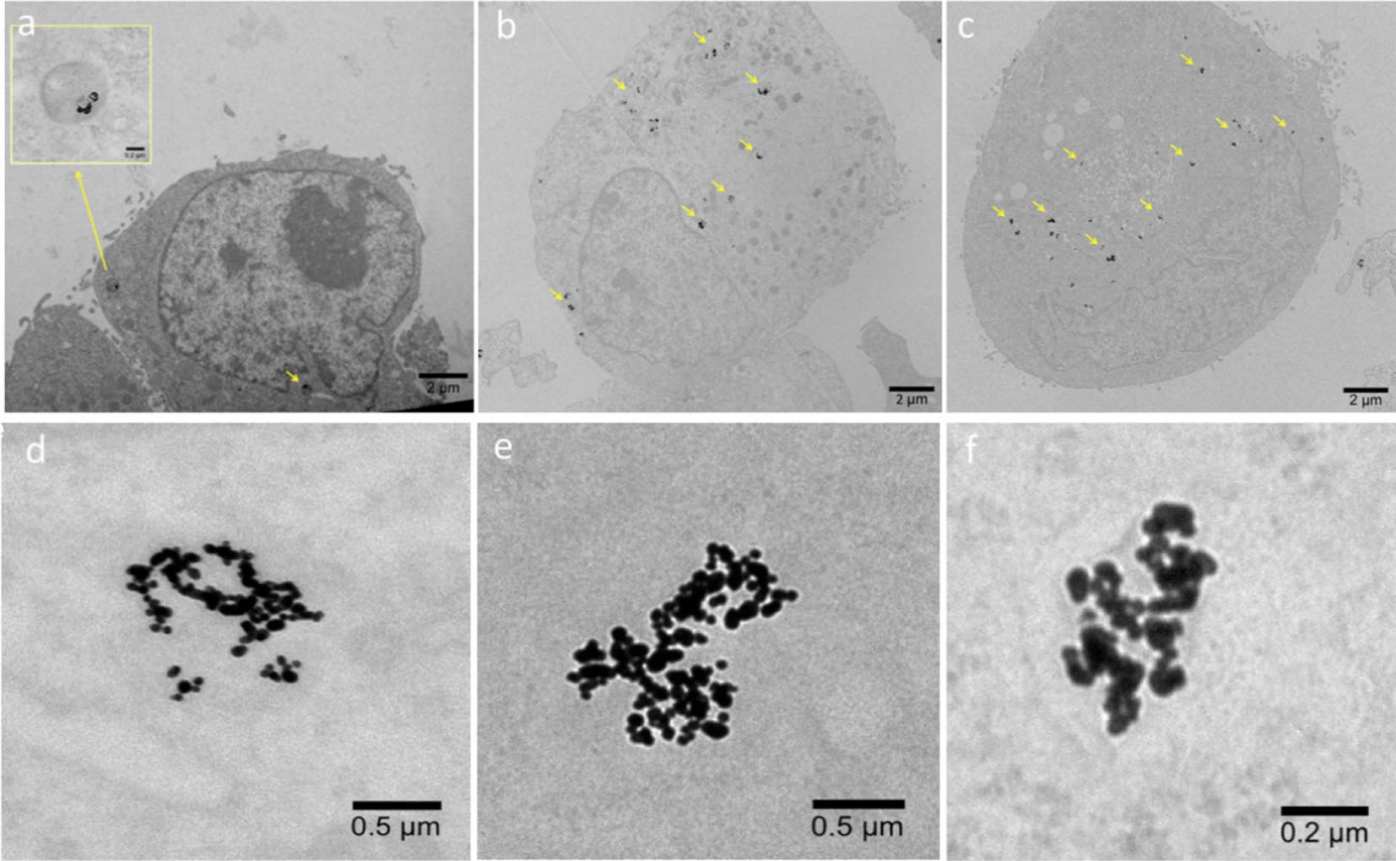
TEM images of MiaPaCa-2 cells after 18 h incubation with P(PEGMA)100 coated GNShells (**a**,**c**) across the cytosol (**a**–**c**) and at higher magnification (**d**–**f**).

**Figure 5:**
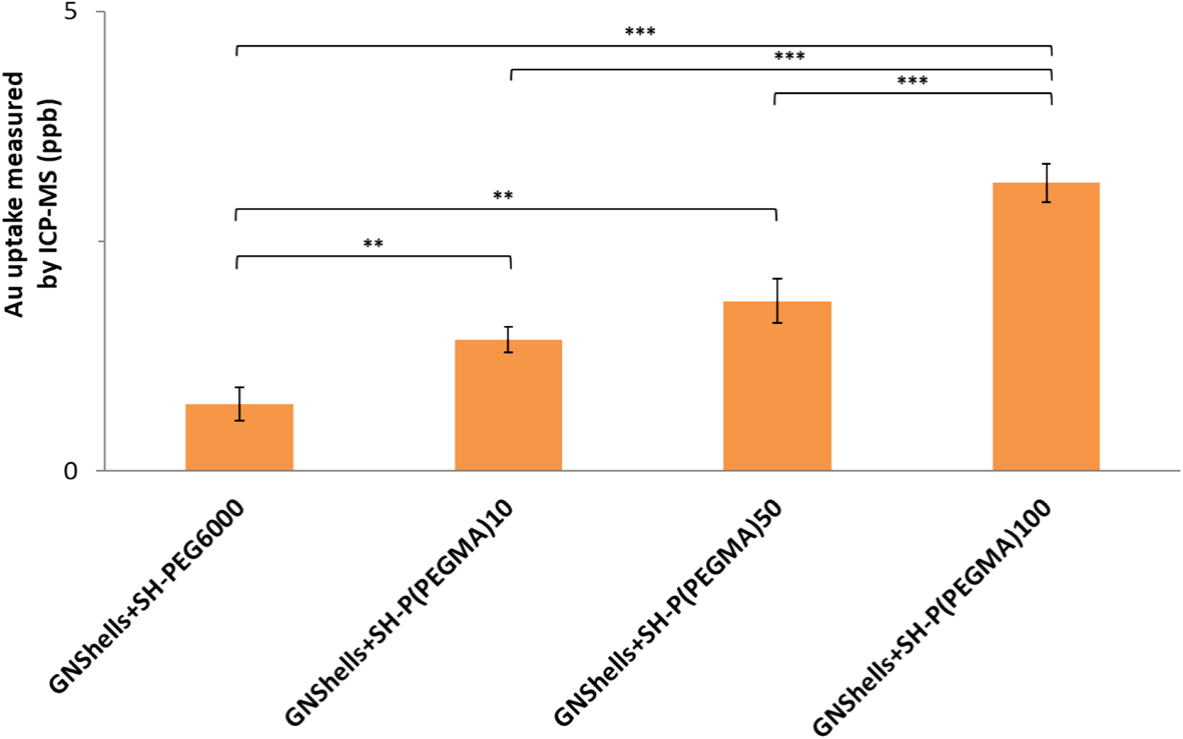
Polymer coated GNShell uptake as measured by ICP-MS showing increasing cellular gold uptake with PEGMA polymer chain length. Error bars are based on standard deviations of three experiments at each data point. Asterisks denote statistical significance as *p < 0.01–0.05, **p < 0.001–0.01, ***p < 0.0001–0.001.

It was also found that the P(PEGMA)10, P(PEGMA)50, and P(PEGMA)100) coated samples had significantly lower EC_50_ compared to the PEG (PEG6000) coated samples; the P(PEGMA)100 coated sample performed better and had an EC_50_ marginally higher than the native drug. Interestingly, the EC_50_ values of the P(PEGMA) GNShells decreased proportionally to the molecular weight of the polymers as shown in Table 3 and hence, given that the parent GNShells were uniform across all batches, and the drug loading efficiency was relatively similar across the samples, we hypothesized that the polymers could modulate the cellular uptake of the GNShells which could explain the observed pattern of the EC_50_ values. Initially, we examined qualitatively the cellular uptake by TEM (Fig. 4). It was found GNShells were uptaken in the form of clusters in lysosomes which were uniformly spread across the cytosol. To obtain a quantitative estimation of the uptake rates per sample, we performed ICP-MS analysis on MiaPaCa-2 cells that had previously been exposed with equal amounts of polymer-coated GNSHells (Fig. 5). Indeed, it was found that the lower gold signal was observed in the PEG6000 coated GNshells while there was a proportional increment of the gold signal in respect to the molecular weight of the P(PEGMA) coated samples with the P(PEGMA)100 coated sample having the highest cellular uptake. These results fully corroborate with the EC_50_ values and verify our hypothesis that the cytotoxicity of the GNshells is causatively proportional with the cellular uptake. Finally, given their higher uptake rates, we shortlisted the P(PEGMA)100-coated GNSHells samples for all further in vitro studies.

**Table 3:** Effect of laser irradiation time on the EC_50_ of the GNShells + SH-P(PEGMA)100 + Gem nanoformulations.

Cell line	Treated with	Incubation time (h)	EC50 (μM)
MiaPaCa-2 (1 × 10^4^ cells/well)	Free Gem (10 min. irradiation)	72	0.74 ± 0.14
	GNShells + SH-P(PEGMA)100 + Gem (No irradiation)		1.76 ± 0.31
	GNShells + SH-P(PEGMA)100 + Gem (1 min. irradiation)		1.63 ± 0.21
	GNShells + SH-P(PEGMA)100 + Gem (5 min. irradiation)		1.20 ± 0.27
	GNShells + SH-P(PEGMA)100 + Gem (10 min. irradiation)		0.54 ± 0.17

The effect of laser treatment on MiaPaCa-2 cells was studied after incubating them with the nanomedicines for 48 and 72 h followed by laser treatment for 1, 5, and 10 min at 0.9 W cm^−2^ (Figure S7 and S8). First, a control experiment comprising Gem only was conducted which showed a concentration dependent cytotoxicity against MiaPaCa-2 cells after 48 h and 72 h. However, there was no significant difference in cell viability with laser treatment in any of the timeframes tested indicating that the laser alone could not enhance the effectiveness of chemotherapy treatment alone (Figure S7). This was also reflected in the EC_50_ values of Gem which was remained unaltered with laser treatment (Table S5). Next, the photothermal effect of polymer-coated GNShells on cytotoxicity was evaluated at equivalent Gem concentrations ranging from 0.001 μM to 1 μM, at 48 and 72 h. The viability of polymer-conjugated GNShell incubated cells was found to be negligible to concentrations up to 10 μM even at irradiation for 10 min for both 48 and 72 h incubation groups (Figure S8). The photothermal effect started to be detectable at 100 μM where statistically significant differences in cell viability could be observed between different irradiation times. This is explained by the fact that the photothermal effect is proportional to the polymer-conjugated GNShell concentration as well as the irradiation time. In addition, the photothermal effect on cell viability was more pronounced in the 72 h group of cells, but still not as lethal to reduce cell viability below 50%.

Having established the toxicity profiles of the individual components we then conducted dose dependent laser treatment experiments with the Gem-loaded P(PEGMA)100-functionalized GNShells. First, it was observed that the laser treatment for 1 and 5 min did not induce significant reduction of the cell viability across all doses (Figure S9). However, it was observed that the 10-min irradiation protocol had a profound effect in all tested concentrations which was noticeable even in the cells treated with the most dilute concentration of 0.001 μΜ P(PEGMA)100-functionalized GNShells + Gem. A gradual reduction of the cell viability was observed with increasing the concentration of the GNShells in both 48 and 72 h P(PEGMA)100-functionalized GNShell + Gem incubated groups. Remarkably, the cell viability reduction dropped to as low as ca. 13% in the 10-min laser treated cells that had been incubated for 72 h with GNSHells. The observed trends in different treatments can be compared by comparing the respective EC_50_ values for each treatment group across the cells which were incubated for 72 h as the cytotoxic effects were more pronounced (Table 3). The EC_50_ of Gem loaded GNShells is 2.3 times higher compared to the parent drug and only marginally improves when the laser is used for 1 or 5 min. However, the EC_50_ is restored and is even slightly improved (0.54 μM and 0.74 μM, for GNShells + Gem and free Gem, respectively, Table 3) with 10 min laser irradiation.

**Figure 6:**
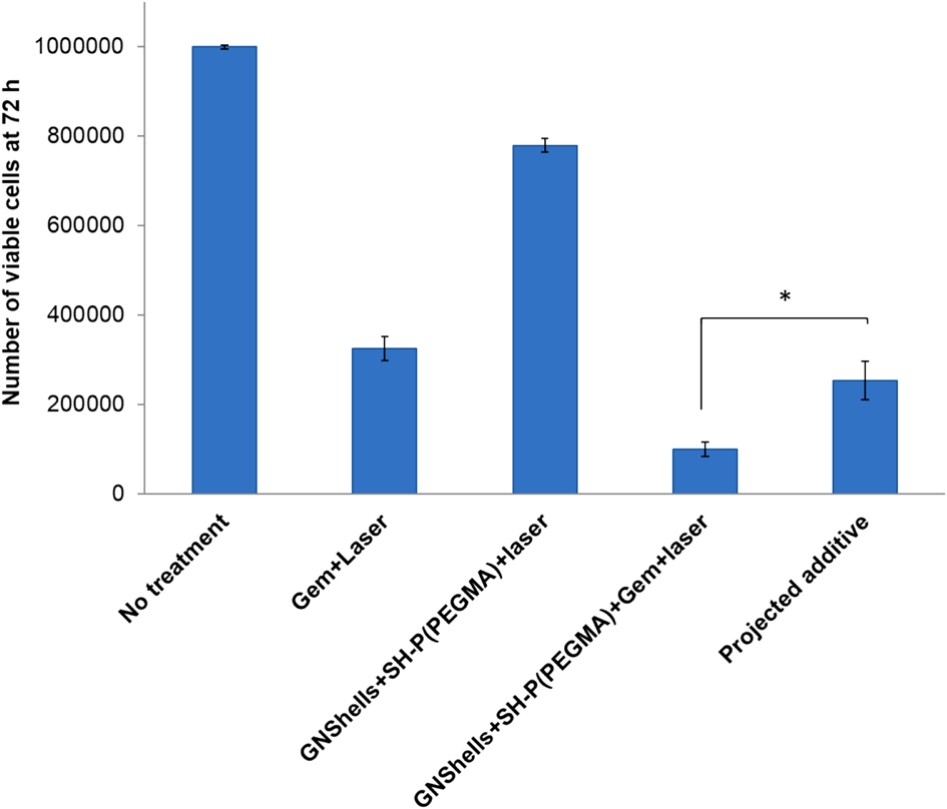
Comparison of cell count at 72 h incubation of MiaPaCa-2 cells untreated and treated with various controls at fixed concentration of GEM at 100 μmol/L. Laser dose was λ = 640 nm, 0.9 W/cm^2^, for 60 min. The calculated projected additive was used as an evaluation index. Error bars represent standard deviations of three samples at each data point. Asterisk denotes statistical significance *p < 0.01).

Given the minimum toxicity profiles of Gem-free GNShells with and without laser irradiation, and also the relatively low amount of Gem which is loaded in the GNSHells (compared to the free control Gem samples), we believe that the marked phototoxicity should be attributed to synergistic photothermal and drug-induced effects. To verify possible synergism the effect of either chemotherapy or phototherapy treatments was determined according to the additive therapeutic interaction of independent treatments as an evaluation index. The final population of MiaPaCa-2 cells after additive interaction (so called projected additive, *P*_*additive*_) was calculated by multiplying the fractions of surviving cells for each independent treatment as (f), that is *P*_*additive*_ = (*f*_*chemotherapy*_ × *f*_*phototherapy*_) × *P*_*0*_*,* where *P*_*0*_ is the starting population (Eq. 3). We considered three treatment groups, namely, free Gem + laser, GNShells + laser, and Gem loaded GNShells + laser. *P*_*additive*_ is the cell viability expected if there was no interplay between chemotherapy and phototherapy and was found to be significantly higher compared to the actual surviving cells exposed to the combinational photo-chemotherapy protocol (Gem loaded GNShells + laser)(*p < 0.01–0.05) which clearly confirms the synergistic activity of the photo- and chemo- therapeutic components of the nanoformulations (Fig. 6). Some interesting conclusions emerge from the synergism analysis and the EC_50_ comparative analysis: 1. Polymer coating indeed plays a role in the observed cytotoxicity as previously discussed and 2. EC_50_ enhancement is not dependent on drug release rate as we do not observe significant dependence of the drug release rates by laser treatment but rather on the impact of mild hyperthermia on a. nanoparticles’ uptake and b. presumably on increased and persistent thermosensitization of cells by GNSshells. The latter effect seems to be critical given that we apply laser treatment 24 h post GNShell incubation with cells implying that Gem is virtually fully released in the culture medium and the cytosol.

**Figure 7:**
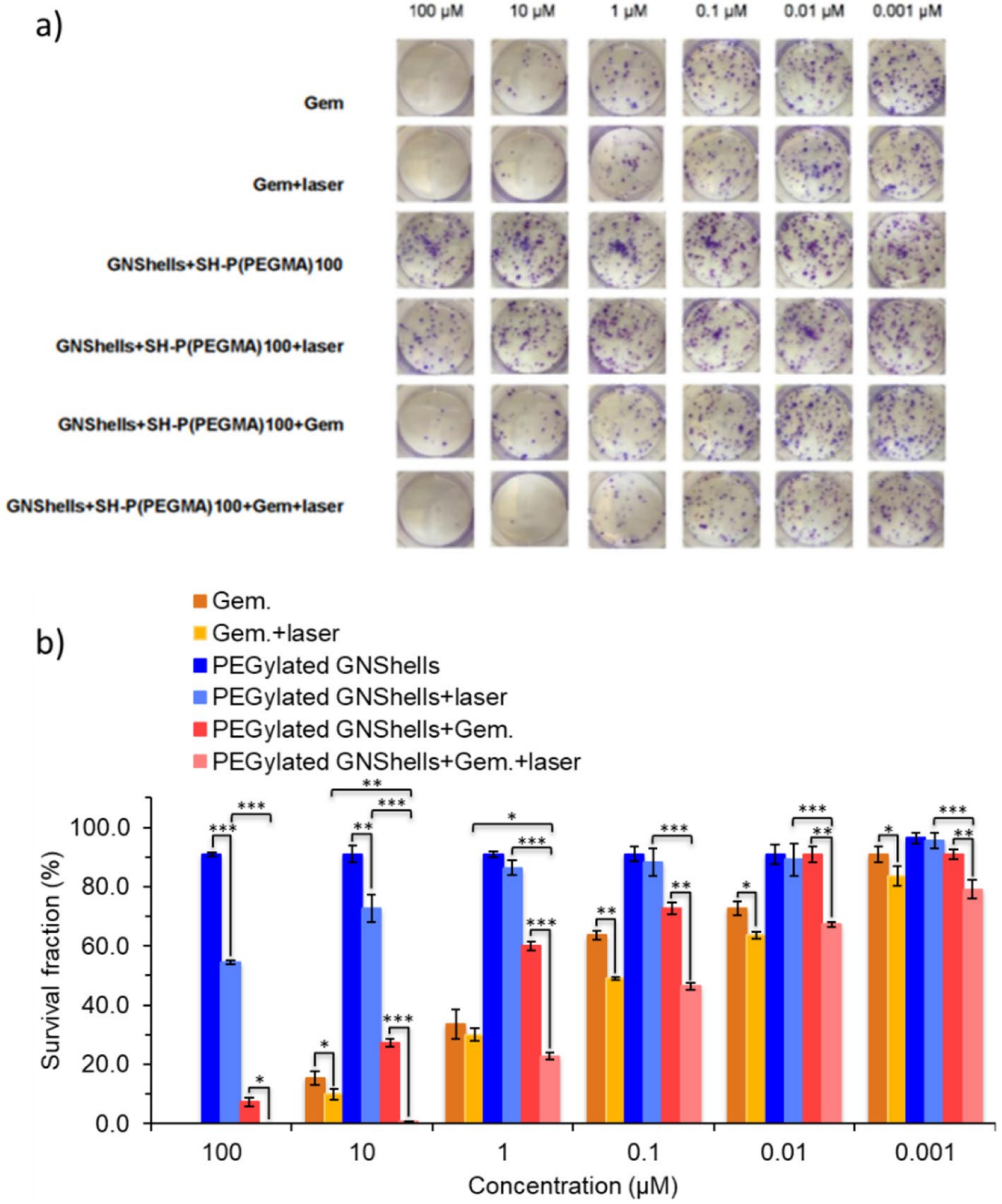
(**a**) Digital photographs of the colonogenic assay of MiaPaCa-2 cells performed with the incubation of cells with free Gem, GNShells + SH-P(PEGMA)100 and GNShells + SH-P(PEGMA)100 + Gem with and without laser irradiation (0.9 W/cm^2^, 60 min) as a function of Gem concentration and (**b**) the percentile survival fraction of MiaPaCa-2 cells post-treatment with and without laser irradiation as a function of Gem concentration after 14 days. Error bars are based on standard deviations of three samples at each data point. Asterisks denote statistical significance as *p < 0.01–0.05, **p < 0.001–0.01, ***p < 0.0001–0.001.

Finally, a clonogenic assays was conducted to evaluate the longer-term cytotoxic activity of the nanoformulations compared to the parent drug by monitoring the proliferative ability of the surviving cells for 14 days post treatments (Fig. 7). Formation of colonies is indicative of the ability of cells to retain their reproductive capacity to reproduce a large colony or a clone. After counting the number of colonies (n = 3), the surviving fraction (SF%) of MiaPaCa-2 cells was calculated for a series of different concentrations of all the three treatment groups with and without laser irradiation (Fig. 7). Visual inspection of the colonies clearly showed that the Gem control and Gem-rich formulations with or without laser irradiation showed a decreasing SF as the concentration of GEM of the nanoformulations increased (Fig. 7). Expectedly, the P(PEGMA)100 coated GNShells samples did not exert any noticeable cytotoxic effects at any concentrations but did exhibit marginal colony spread inhibition at high concentrations with laser treatment.

The observed trends could be quantified to clearly demonstrate the effect of Gem and laser combination on colony spreading inhibition. Interestingly, the P(PEGMA)100 coated GNShells with Gem could retain a SF below 50% at concentrations as low as 0.1 μM when treated with laser, clearly outperforming non-irradiated samples (*p < 0.01–0.05, **p < 0.001–0.01); at higher concentrations, the SF reached minuscule percentiles close to zero SF.

## Conclusion

We demonstrated a potent polymer-gold hybrid nanoformulation for the delivery of gemcitabine accompanied by confined hyperthermia activation via red laser irradiation against pancreatic cancer cells. It was shown that end-group polymer functionalization allows for the preparation of nanoformulations with different surface properties which ultimately impact the biological performance in respect to the cellular uptake and drug release profiles. It seems that even though PEG and P(PEGMA) polymers share structural similarities, the backbone rigidity of the latter seems to alter the overall properties of the PEG counterpart as we noticed marked differences both in formulation experiments and in the in vitro studies. More importantly, it was shown that the EC_50_ of the parent drug could be diminished when loaded on the GNShells but could be restored and even improved at confined areas where irradiation takes place. The combinational effect of the drug and local hyperthermia resulted in highly toxic potency which was considerably higher than the parent drug and also had longer lasting effect. Potentially, this implies that under an in vivo scenario, it may be possible to locally modulate the EC_50_ of the drug precisely at the site of interest with the use of laser-induced hyperthermia. In principle, this laser controlled modulation of the EC_50_ efficacy of the nanoformulations could lead to more potent therapeutics for pancreatic cancer in that the maximum tolerated dose of the drug could be increased to harness the augmented toxicity spatially only at the tumor areas in concert with photothermal treatment.

## Supplementary Information

Supplementary Information
