# Supplementary material for: Polymer coated gold nanoshells for combinational photochemotherapy of pancreatic cancer with gemcitabine
Source: Sci Rep. 2021 Apr 30;11:9404. doi: 10.1038/s41598-021-88909-x (PMC8087785; doi:10.1038/s41598-021-88909-x)
Supplement: Supplementary file 1 — Supplementary Information [file 41598_2021_88909_MOESM1_ESM.pdf]

Supporting Information

Polymer Coated Gold Nanoshells for Combinational Photochemotherapy of Pancreatic Cancer with Gemcitabine

Mina Emamzadeh[1], and George Pasparakis*[1,2]

[1] University College London, School of Pharmacy, London WC1N 1AX

[2] Current address: University of Patras, Department of Chemical Engineering

**Correspondence to: gpasp@chemeng.upatras.gr*

Table S1. The thiolated PEG/PEGMA polymers used for PEGylation of GNShells.

Polymers	M_n *(g mol^{-1})*	C (mg mL^{-1})
SH-PEG6000	6000	1
SH-P(PEGMA)10	4 200	0.65
SH-P(PEGMA)50	14 900	0.2
SH-P(PEGMA)100	28 300	0.1

Table S2. Experimental design of *in vitro* chemotherapy, phothothermal, and chemothermal treatments.

**Drug/nanoformulations**	**Dose of drug/ nanoformulations**	**Laser exposure time (min)**
**Free Gem.**	ranging from 0.001 to 100 µmol L^{-1}	1<br>5<br>10
**GNShells+SH-P(PEGMA)100+Gem.**		
**GNShells+SH-P(PEGMA)100**	GNShells+SH-P(PEGMA)100+Gem equivalent doses	
Note: For irradiating cell culture experiments, the treated cells in 96-well plates exposed to red laser (640 nm, 0.9 W cm^{-2}) for 1 min (169J 60s 0.32cm^{-2}), 5 min (844J 300s 0.32cm^{-2}) and 10 min (1687J 600s 0.32cm^{-2}).		

Table S3. GNShells+SH-P(PEGMA)100+Gem (OD = 1).

D_h (nm)	*λ (nm)*	*C (particle/mL)*	*Molarity (M)*	*Mass of gold (μg/mL)*
70.81 ± 1.83	640	~1.8×10^{11}	25×10^{-5}	85

Notes: The original concentration of as-prepared GNShells was estimated as about 0.25 mM according to the initial concentration of gold precursors.

Table S4. X-ray photoelectron spectra of gold (a) and Nitrogen (b) of the Gem-loaded GNShells.

	a	b
**GNShells+ SH-PEG6000 +Gem**	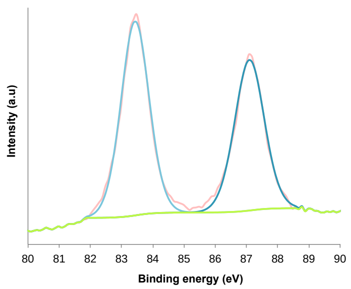	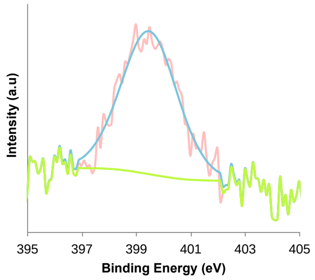
**GNShells+ SH-P(PEGMA)10 +Gem**	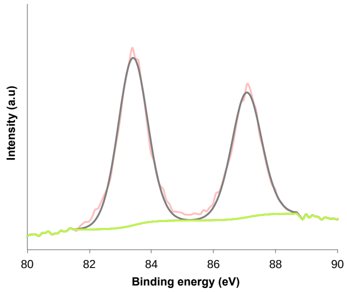	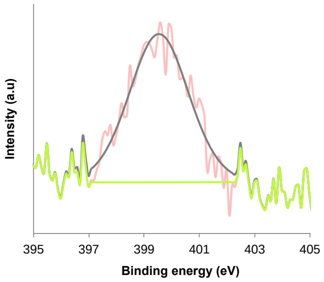
**GNShells+ SH-P(PEGMA)50 +Gem**	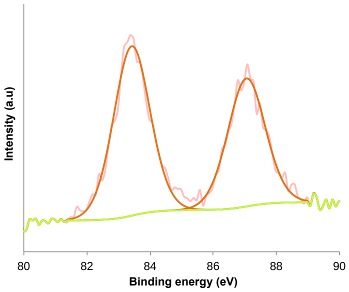	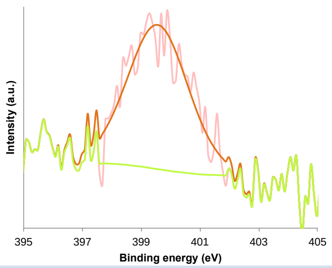
**GNShells+ SH-P(PEGMA)100 +Gem**	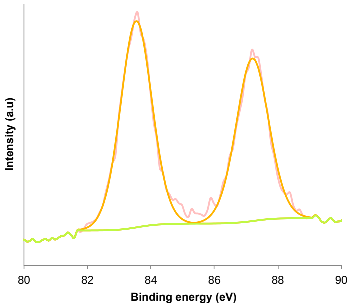	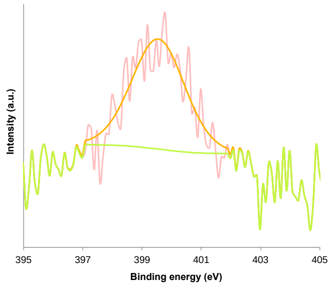

Table S5. EC_{50} (µM) of Gem against MiaPaCa-2 cells before and after irradiation (λ = 640 nm, 0.9 W cm-2, 1-5-10 min). All the MTT experiments were performed in triplicate and the variation in EC_{50} are shown as mean±SD.

Cell line	Treated with	Incubation time (h)	EC_{50} (µM)
**Miapaca-2 ($1x10^4$ cells/well)**	Gem. (No irradiation)	72	0.72±0.04
	Gem (1 min irradiation)		0.66±0.05
	Gem (5 min irradiation)		0.68±0.01
	Gem (10 min irradiation)		0.74±0.14

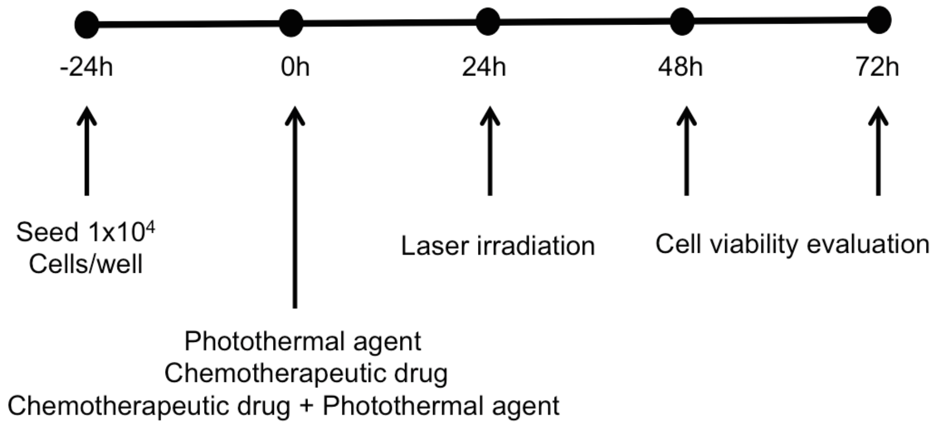

Scheme S1. Experimental design. Illustration of the experimental design, with each treatment replicated in three independent experiments.

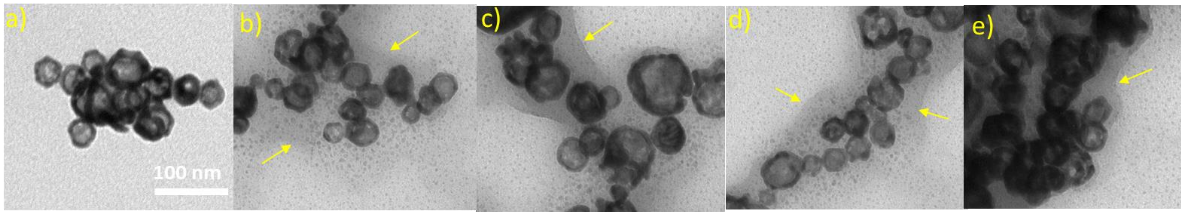

Figure S1. TEM images of a) non-coated GNshells, and coated with b) PEGMA10, c) P(PEGMA)50, d) P(PEGMA)100 and e) PEG6000. Yellow arrows depict the presence of the polymeric layer.

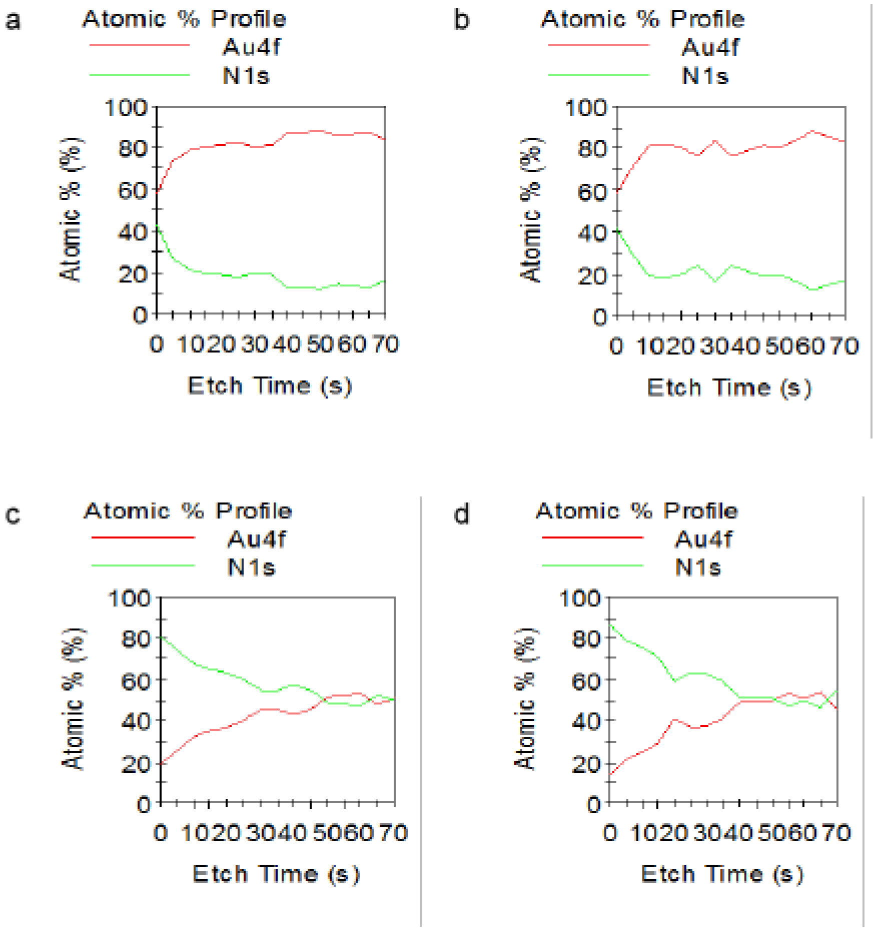

Figure S2. XPS composition-depth profiles (atomic percentage against etch time (s)) of GNShells-SH-PEG6000+Gem (a), GNShells-SH-P(PEGMA)$_{10}$+Gem (b), GNShells-SH-P(PEGMA)$_{50}$+Gem (c), and GNShells-SH-P(PEGMA)$_{100}$+Gem.

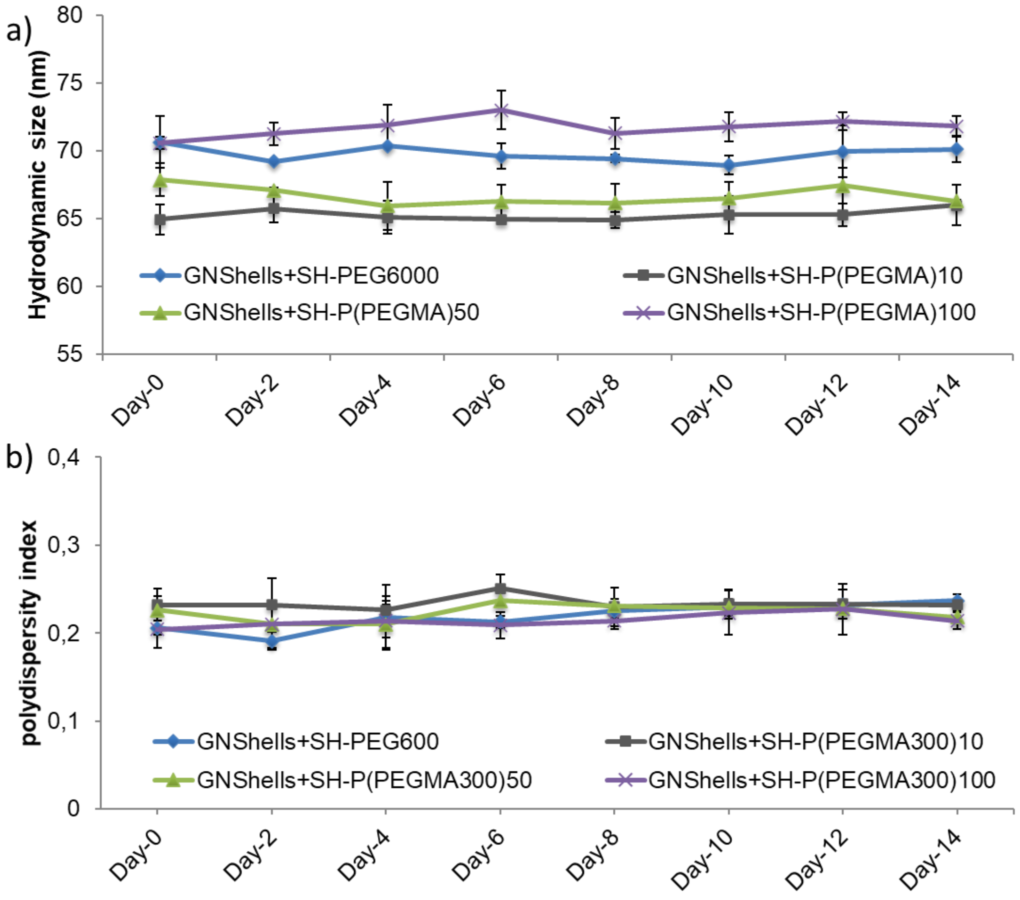

Figure S3. a) Colloidal stability of polymer coated GNShells for 14 days by monitoring their hydrodynamic size in a) and the PDI in b). The data presented as the mean value of three experiments.

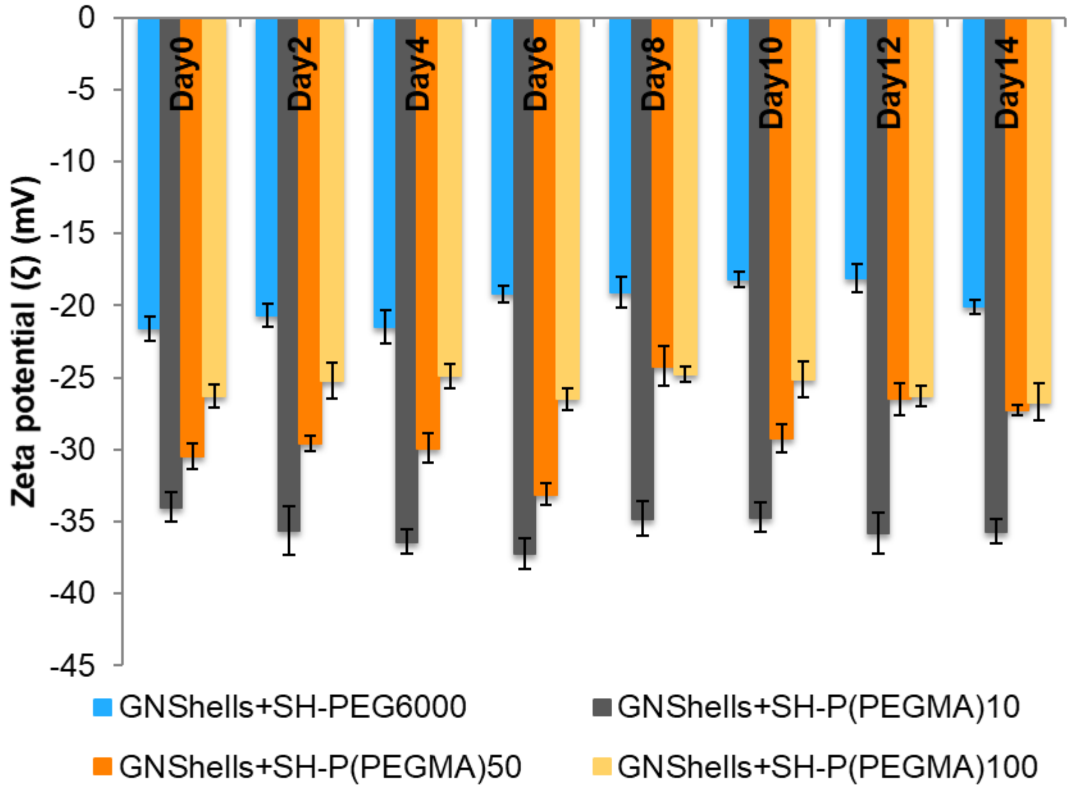

Figure S4. ζ-potential values of polymer coated GNSHells for 14 days. The data presented as the mean value of three experiments.

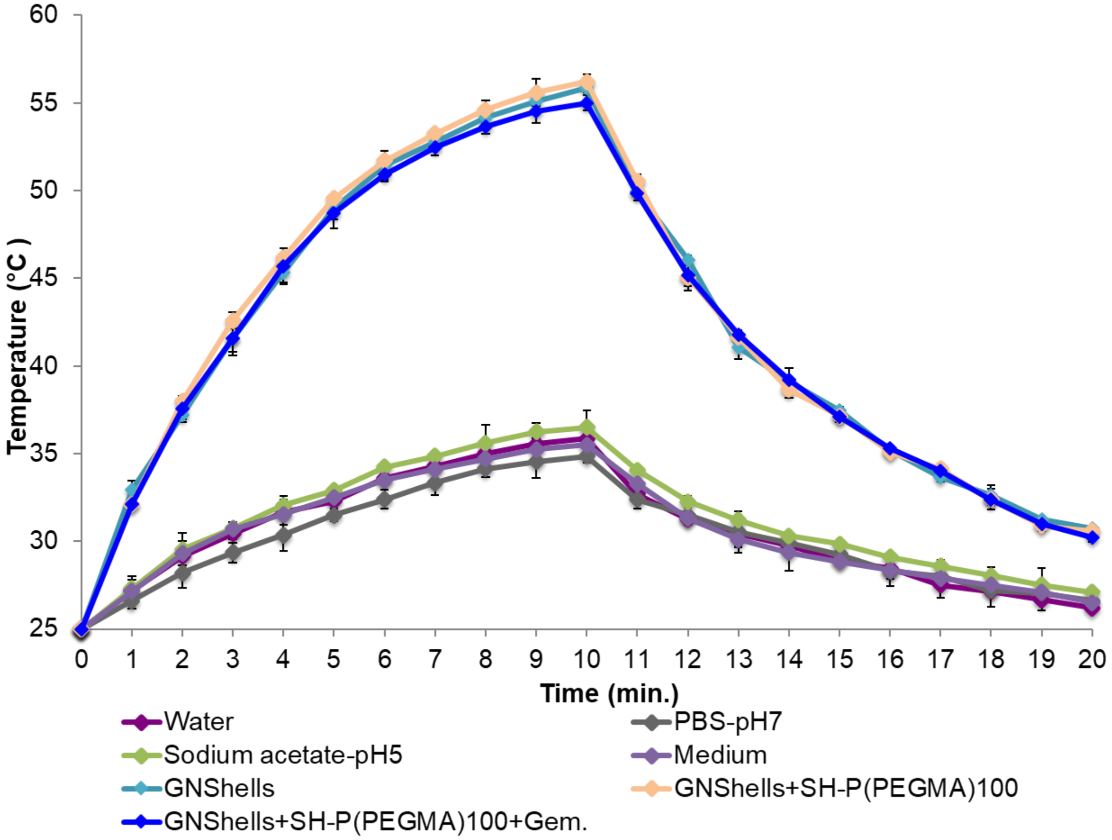

Figure S5. Temperature increase of dH_2O, PBS, sodium acetate buffer, cell culture medium, GNSHells coated with $P(PEGMA)_{100}$+GEM induced by CW red laser irradiation (0.9 W/cm^{-2}, 640 nm, 10 minutes) followed by monitoring of the cooling effect after the laser was turned off (n=3).

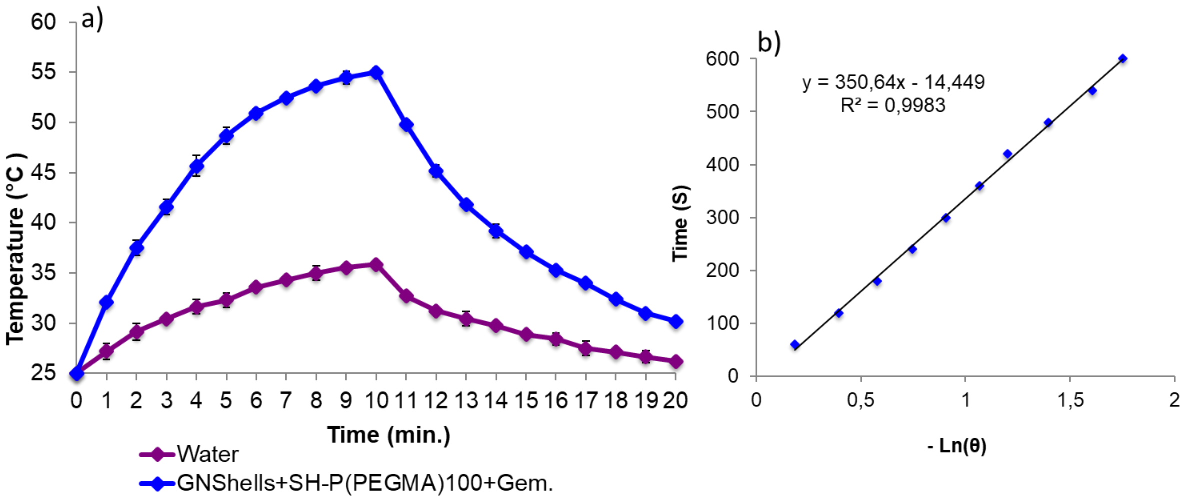

Figure S6. a) Laser induced temperature increase by dH_2O (as control) and GNShells+SH-P(PEGMA)100+GEM, irradiated with 640 nm red CW laser (0.9 W/cm^{-2}), and b) Plot of the negative logarithm of temperature (θ) as a function of time after turning off the laser.

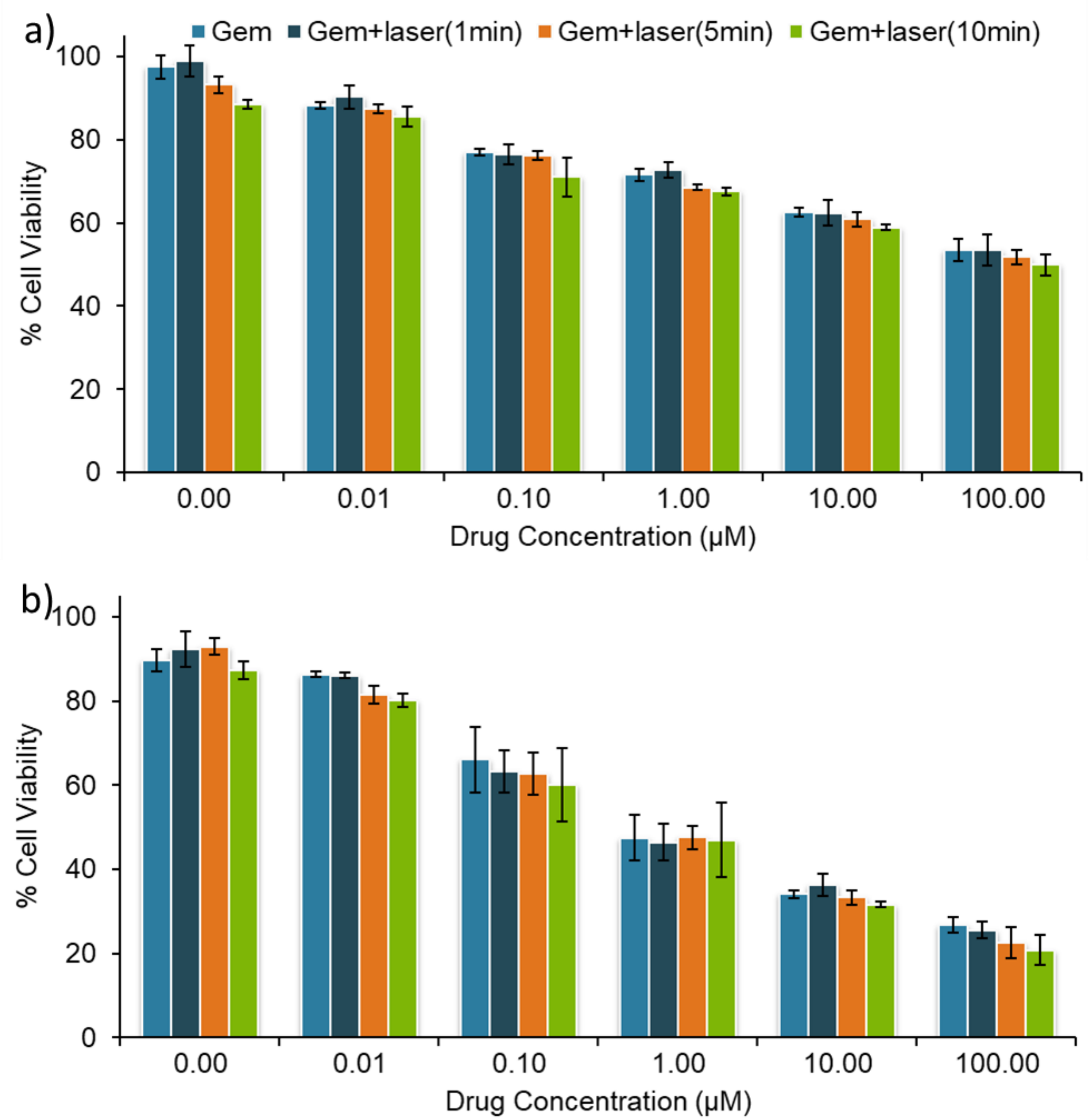

Figure S7. Cell viability of Gem and Gem plus laser irradiation (λ=640 nm, 0.9 W cm^{-2}, 1, 5, and 10 minutes) for MiaPaCa-2 cells as a function of Gem concentration after a) 48 h and b) 72 h incubation. The data represented as the mean of three experiments.

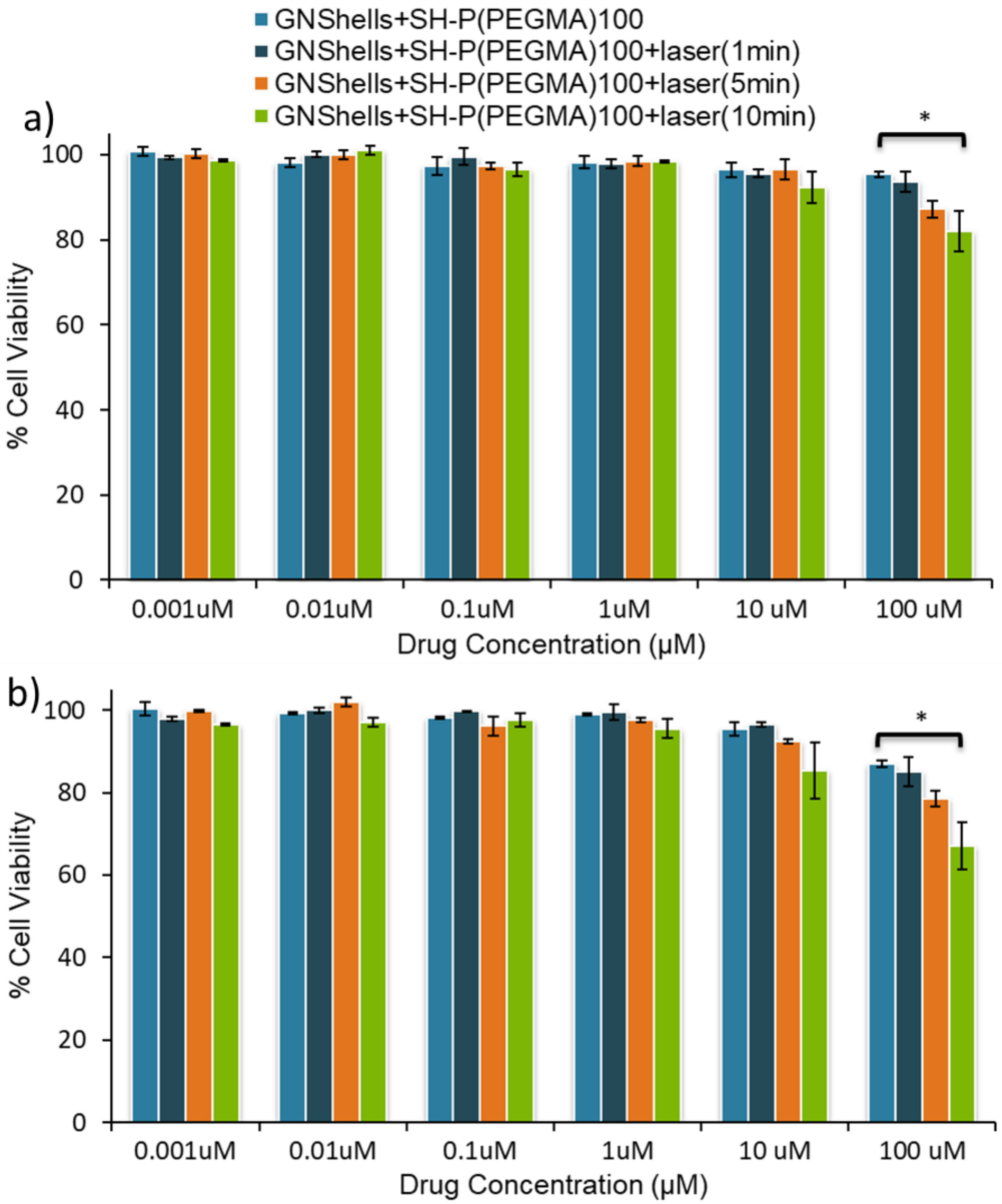

Figure S8. Cell viability of GNSHells+SH-P(PEGMA)100 and GNSHells+SH-P(PEGMA)100 plus laser irradiation (λ=640 nm, 0.9 W cm^{-2}, 1, 5, and 10 minutes) for MiaPaCa-2 cells as a function of GEM concentration after a) 48 h and b) 72 h. error bars are based on standard deviations of three samples at each data point. Asterisks denote statistical significance from control (**p*<0.01-0.05).

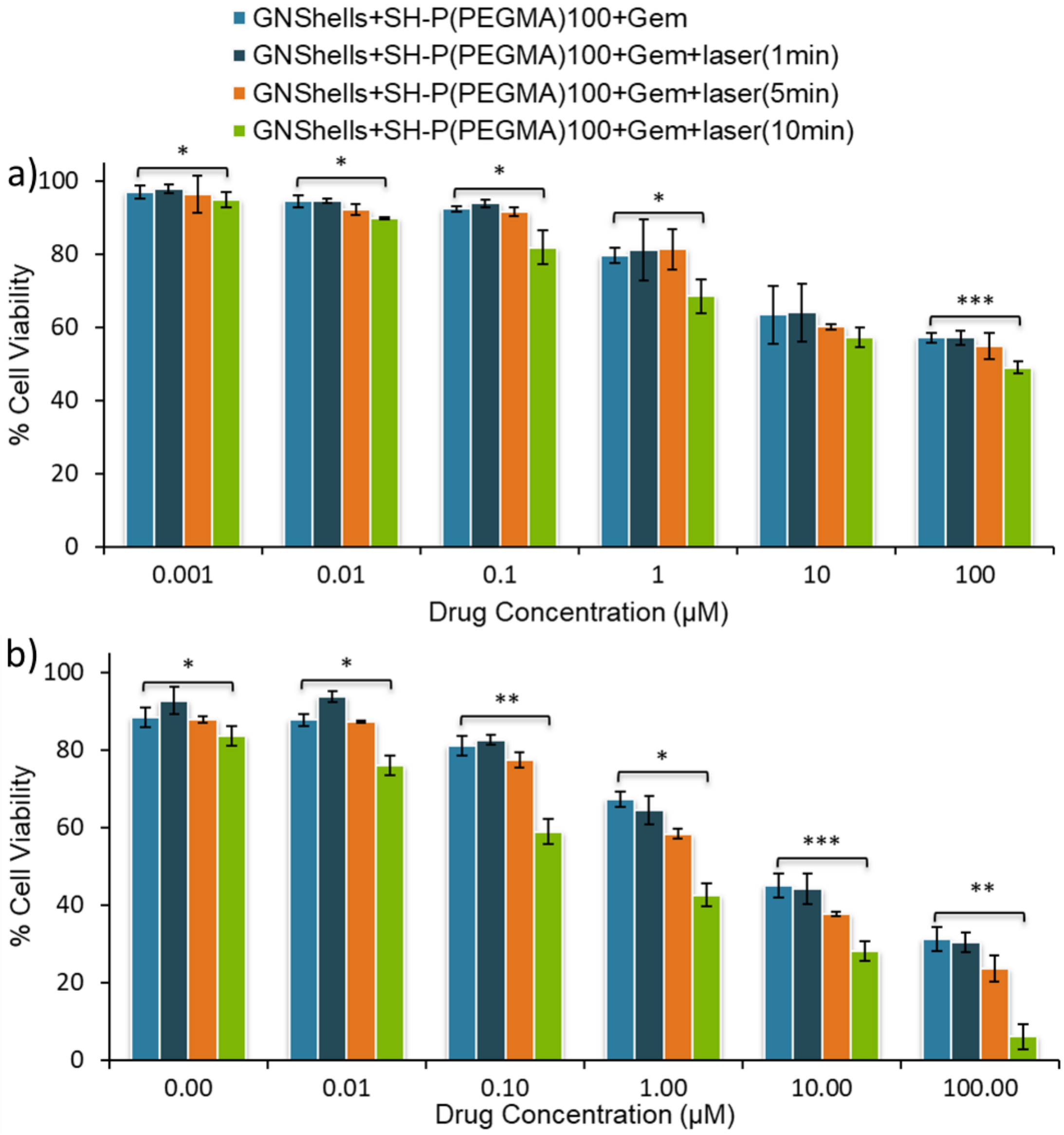

Figure S9. Cell viability of GNSHells+SH-P(PEGMA)100+Gem and GNSHells+SH-P(PEGMA)100+Gem plus laser irradiation (λ=640 nm, 0.9 W cm^{-2}, 1, 5, and 10 minutes) for MiaPaCa-2 cells as a function of GEM concentration after a) 48 h and b) 72 h.
